# The CC-NB-LRR-Type *Rdg2a* Resistance Gene Confers Immunity to the Seed-Borne Barley Leaf Stripe Pathogen in the Absence of Hypersensitive Cell Death

**DOI:** 10.1371/journal.pone.0012599

**Published:** 2010-09-10

**Authors:** Davide Bulgarelli, Chiara Biselli, Nicholas C. Collins, Gabriella Consonni, Antonio M. Stanca, Paul Schulze-Lefert, Giampiero Valè

**Affiliations:** 1 Genomic Research Center, CRA-GPG, Fiorenzuola d'Arda, Italy; 2 Department of Plant Microbe Interactions, Max Planck Institute für Züchtungsforschung, Köln, Germany; 3 Australian Centre for Plant Functional Genomics, School of Agriculture Food and Wine, University of Adelaide, Glen Osmond, Australia; 4 DiPROVE, University of Milan, Milano, Italy; Ecole Normale Superieure, France

## Abstract

**Background:**

Leaf stripe disease on barley (*Hordeum vulgare*) is caused by the seed-transmitted hemi-biotrophic fungus *Pyrenophora graminea*. Race-specific resistance to leaf stripe is controlled by two known *Rdg* (*R*esistance to *Drechslera graminea*) genes: the *H. spontaneum*-derived *Rdg1a* and *Rdg2a*, identified in *H. vulgare*. The aim of the present work was to isolate the *Rdg2a* leaf stripe resistance gene, to characterize the *Rdg2a* locus organization and evolution and to elucidate the histological bases of *Rdg2a*-based leaf stripe resistance.

**Principal Findings:**

We describe here the positional cloning and functional characterization of the leaf stripe resistance gene *Rdg2a*. At the *Rdg2a* locus, three sequence-related coiled-coil, nucleotide-binding site, and leucine-rich repeat (CC-NB-LRR) encoding genes were identified. Sequence comparisons suggested that paralogs of this resistance locus evolved through recent gene duplication, and were subjected to frequent sequence exchange. Transformation of the leaf stripe susceptible cv. Golden Promise with two *Rdg2a*-candidates under the control of their native 5′ regulatory sequences identified a member of the CC-NB-LRR gene family that conferred resistance against the Dg2 leaf stripe isolate, against which the *Rdg2a*-gene is effective. Histological analysis demonstrated that *Rdg2a*-mediated leaf stripe resistance involves autofluorescing cells and prevents pathogen colonization in the embryos without any detectable hypersensitive cell death response, supporting a cell wall reinforcement-based resistance mechanism.

**Conclusions:**

This work reports about the cloning of a resistance gene effective against a seed borne disease. We observed that *Rdg2a* was subjected to diversifying selection which is consistent with a model in which the *R* gene co-evolves with a pathogen effector(s) gene. We propose that inducible responses giving rise to physical and chemical barriers to infection in the cell walls and intercellular spaces of the barley embryo tissues represent mechanisms by which the CC-NB-LRR-encoding *Rdg2a* gene mediates resistance to leaf stripe in the absence of hypersensitive cell death.

## Introduction

Leaf stripe disease on barley (*H. vulgare*) is caused by the seed-transmitted hemi-biotrophic fungus *Pyrenophora graminea* (anamorph *Drechslera graminea*) [(Rabenh. ex. Schlech.) Shoemaker]. The disease causes severe yield reductions at high infection rates, especially in organic farming systems [1], [2]. The fungal mycelia survive in seeds between the parenchymatic cells of the pericarp, and in the hull and the seed coat, but not in the embryo [3]. During seed germination, the hyphae begin to grow intercellularly within the coleorhizae, and then into the embryo structures, the roots and scutellar node, to establish infection in the seedling. During this first colonization phase the pathogen behaves as a biotroph and degrades host cell walls using hydrolytic enzymes without causing cellular necrosis [3]–[5]. Once infection spreads into the young leaves, growth switches to a necrotrophic phase with the production of a host-specific glycosyl toxin [6] that causes longitudinal dark brown necrotic stripes between the leaf veins, as well as spike sterility. Spores produced on the infected leaves of susceptible plants spread to infect nearby plant spikes.

Race-specific resistance to leaf stripe is controlled by two known *Rdg* (*R*esistance to *Drechslera graminea*) genes. These genes cause hyphal degeneration in the basal part of the coleorhiza and prevent stripe symptoms from appearing on leaves of young or old plants [3], [7], [5]. *H. spontaneum*-derived *Rdg1a* has been mapped to the long arm of chromosome 2H [8], [9] while *Rdg2a*, identified in *H. vulgare*, has been mapped on the short arm of chromosome 7HS [10]. Both resistance genes have been extensively used in classical breeding, but neither has been cloned. Histological characterization of the *Rdg2a*-dependent resistance response by [5] showed the termination of *P. graminea* growth at the scutellar node and basal region of provascular tissue of the barley embryo. The immune response was associated with cell wall reinforcement through accumulation of phenolic compounds and enhanced transcription of genes involved in reactive oxygen species (ROS) production and detoxification/protection, but no localized programmed cell death (PCD), which is typically seen in race-specific immune responses [11], was apparent.

In this study we describe the cloning of *Rdg2a* and the molecular characterization of the *Rdg2* locus. Bacterial artificial chromosome (BAC) and cosmid libraries respectively derived from barley cvs. Morex (which is susceptible to leaf stripe) and Thibaut (the donor of the *Rdg2a* allele) were used for physical mapping of the locus, leading to the identification of three *Rdg2a* candidates representing sequence-related members of a gene family. Transformation experiments showed that a coiled-coil, nucleotide-binding site, leucine-rich repeat (CC-NB-LRR) encoding gene confers *Rdg2a*-specific resistance. Similar to that of other *R* proteins [12], the RDG2A protein localized to the nucleus and the cytoplasm, while histological analysis confirmed that RDG2A involves cell wall-localized autofluorescence and does not trigger a hypersensitive cell death, consistent with physical/chemical defences mounted by the living cells stopping the intercellularly growing leaf stripe pathogen.

## Results

### Genetic and physical map of the *Rdg2a* locus

The *Rdg2a* locus resides in a chromosome region of high recombination [7], which is a characteristic that would assist in map-based cloning. To investigate the molecular basis of the *Rdg2a*-based *P. graminea* resistance in barley, map-based isolation of *Rdg2a* was initiated by constructing a high resolution genetic map representing 2,800 F_1_ gametes. The locus was delimited to a 0.14 cM marker interval, and a PCR-based marker located 0.07 cM from *Rdg2a* was developed [7].

**Figure 1:**
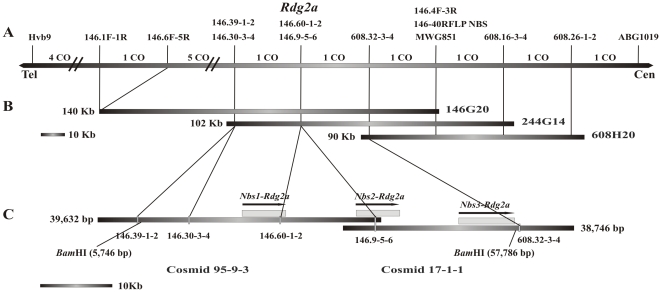
Genetic and physical maps of the *Rdg2a* locus. (A) Genetic map of *Rdg2a*. Crossovers identified in the 1,400 F_2_ plants from a cross between Thibaut (*Rdg2a*) and Mirco [7] are shown at the top (CO). Orientation is indicated by Tel (telomere) and Cen (centromere). (B) Contig of Morex BAC clones. (C) Thibaut cosmid contig and genes at the *Rdg2a* locus. Transcription direction of the genes are indicated by arrows.

Leaf stripe isolate Dg2, which is recognised by *Rdg2a* [10] (Table S1), is virulent on cv. Morex, indicating that this cultivar does not contain a functional *Rdg2a* allele. However, due to the availability of a Morex BAC library [13], we took advantage of this resource for marker development. Utilization of the Morex BAC library for marker development and recessive allele isolation is an approach that was previously used for the isolation of homologues and functional alleles at the *Mla* powdery mildew resistance locus in barley [14]–[16]. Screening of the library with a probe derived from the CAPS marker MWG851 (Methods S1), allowed identification of BAC clones 146G20, 244G14 and 608H20 that were subjected to end sequencing (Methods S1). The 146G20 and 608H20 clones were also subjected to low-pass (0.3-fold) shotgun sequencing and nine additional CAPS, dCAPS or RFLP markers were identified (Figure 1A; Table S2). Two of these (146.60-1-2 and 146.9-5-6) showed complete linkage with *Rdg2a*. These PCR-based markers were tested on the three BAC clones, allowing the markers to be located to sections of the contig (Figure 1B). The estimated size of the 146G20 insert was about 140 kbp. 146.1F-1R and 146.4F-3R markers mapped 0.32 cM apart (9 recombinants out of 2,800 gametes), indicating a genetic to physical ratio of about 440 kb per cM in this *Rdg2a* interval.

To clone the region containing the *Rdg2a* resistance gene, we constructed a genomic cosmid library of the *Rdg2a*-containing cv. Thibaut (Methods S1). Screens using markers 146.9-5-6 and 608.32-3-4 identified the clones 95-3-3 and 17-1-1. Analysis of these two clones with other PCR markers from the region indicated that the clones spanned the *Rdg2a* interval bounded by the closest flanking genetic markers (Figure 1C). The two cosmids which overlapped by 5.9 kb were sequenced, providing a contiguous sequence of 72,630 bp. In BLASTX analyses, the sequenced region was shown to contain three gene models with similarity to plant *R* genes encoding NB-LRR proteins (GenBank accession number HM124452). The three NB-LRR encoding genes were predicted using the AutoPredgeneset tool of the RiceGAAS software (http://ricegaas.dna.affrc.go.jp/, [17]) and designated *Nbs1-Rdg2a*, *Nbs2-Rdg2a* and *Nbs3-Rdg2a* with their relative locations shown in Figure 1C.

RFLP analysis of *Bam*HI digested genomic DNA with probes derived from the NB-LRR genes detected only one fragment of about 50 kbp in the resistant cv. Thibaut and in NIL3876 containing *Rdg2a* (Figure S1), which agreed with the 52 kbp fragment size predicted from the sequence assembly (Figure 1C). In susceptible genotypes, either three fragments were detected (cvs. Mirco and Golden Promise) or a single ∼20 kbp fragment was detected (cv. Morex) indicating large deletion(s) in this last genotype.

### Structure of *Rdg2a* candidate genes

**Figure 2:**
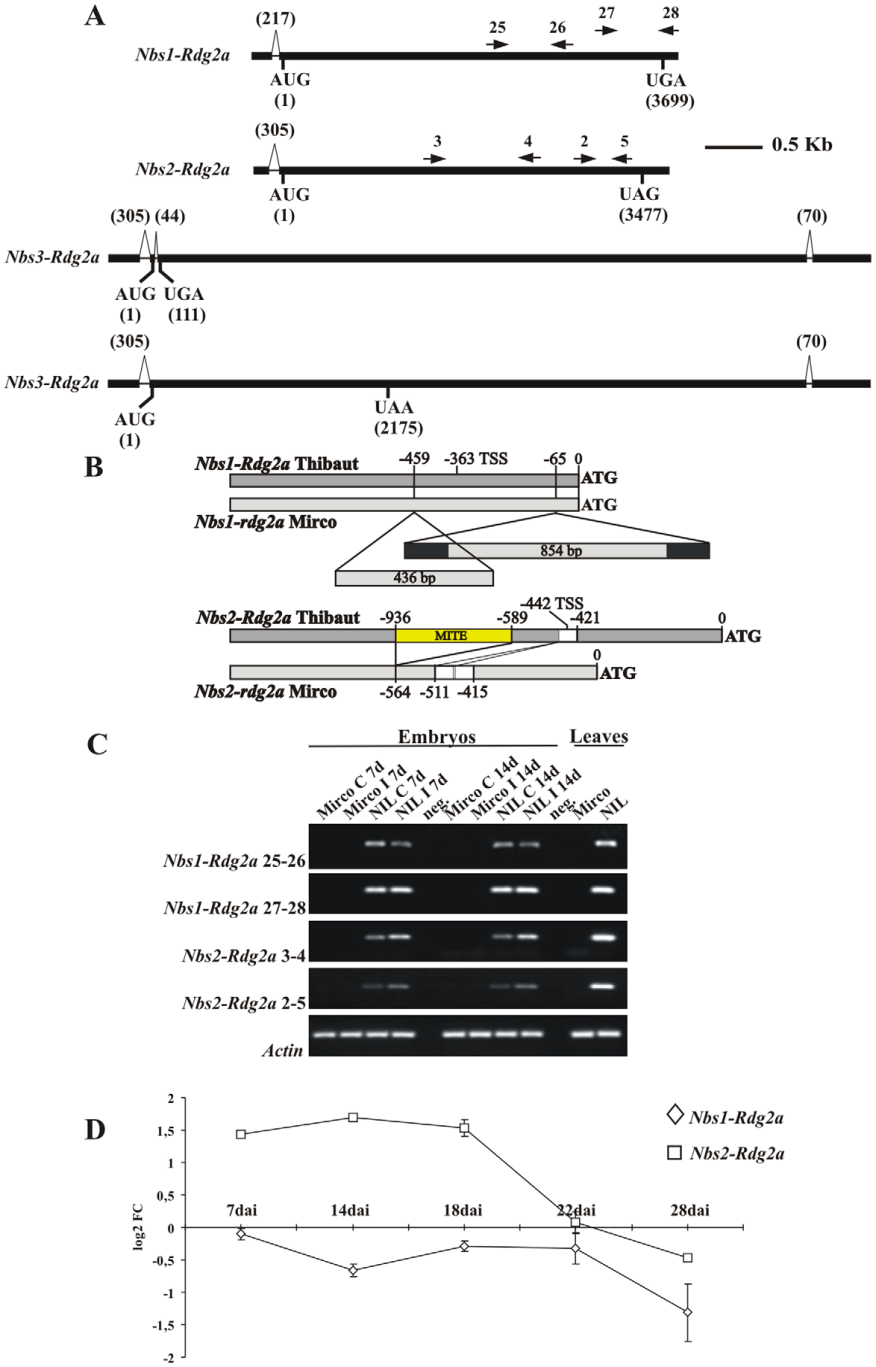
Analysis of *Rdg2a*-candidate gene transcript structure and regulation. (A) *Nbs1-Rdg2a*, *Nbs2-Rdg2a* and *Nbs3-Rdg2a* transcript structures (cv. Thibaut), indicating positions of primers used in transcript quantification. The two transcript types resulting from alternative splicing pattern of *Nbs3-Rdg2a* are indicated. (B) Structural differences between Thibaut and Mirco alleles of *Nbs1-rdg2a* and *Nbs2-rdg2a* genes in 5′ regions. Positions of insertion/deletions relative to the start codon are shown. Filled sections indicate inverted repeats present in an insertion in the Mirco *Nbs1-rdg2a* gene. The *Nbs2-rdg2a* allele comparison illustrates variation for a MITE insertion and a 41–bp direct repeat (open sections). Transcription start sites (TSS) for *Nbs1-Rdg2a* and *Nbs2-Rdg2a* are indicated. (C) Semi-quantitative RT-PCR analysis of the *Rdg2a*-candidate gene expression using gene specific primers. Transcripts were analysed in embryos of the cv. Mirco (*rdg2a*) and NIL3876 (*Rdg2a*) genotypes at two timepoints, after inoculation with *P. graminea* Dg2 (I), or in uninoculated controls (C). Leaves of uninoculated plants were also analysed. Negative controls (neg.) in which DNA was omitted are included. Primers for cv. Thibaut genes were those represented in (A), while primers for amplifying homologous fragments from cv. Mirco were based on the cv. Mirco gene sequences and positioned within 30 bp of the corresponding Thibaut primers. RT-PCR of the barley β-*actin* gene was used as an internal control. (D) Quantitative RT-PCR at 7, 14, 18, 22 and 28 days after pathogen inoculation (dai) for the two *Rdg2a*-candidates in embryos of NIL3876-*Rdg2a*. Values are expressed as log2 fold changes of transcript levels in the inoculated samples with respect to the transcript levels in un-inoculated barley embryos. Error bars represent SD across all RT-PCR replicates (four to six from each of two independent inoculations).

All three *Rdg2a* candidates were found to be transcribed in resistant embryos, and the transcript structures (Figure 2A) were determined by random amplification of cDNA ends (RACE) and RT-PCR. *Nbs1-Rdg2a* and *Nbs2-Rdg2a* had single introns of 217 or 305 bp in the 5′ UTR, and predicted full-length NB-LRR protein products of 1,232 and 1,158 amino acids, respectively. The *Nbs3-Rdg2a* transcript contained a repeat structure, comprising similarity to a full-length NB-LRR protein followed by similarity to part of a NB domain and a full LRR domain (Figure S2). However, the following observations lead us to conclude that *Nbs3-Rdg2a* encodes only predicted truncated proteins. In addition to a 305 bp intron in the 5′ UTR and a 70 bp intron in the 3′ UTR, *Nbs3-Rdg2a* had one 44 bp intron located shortly after the start codon, which was spliced out in only a third (4/12) of the RACE clones analysed. Splicing of the intron causes a frame-shift, resulting in termination after the first 37 amino acids and addition of one novel amino acid (Cys), while retention of this intron results in termination after the first four and a half LRR units (725 amino acids) due a nonsense substitution mutation (Figure 2A). We thought it unlikely that *Nbs3-Rdg2a* encodes a functional resistance protein so we did not pursue it further as an *Rdg2a* candidate.

Apart from the major structural differences, the ORFs of the three genes were 87–90% identical to one another at the DNA level and 81–86% identical and 91–93% similar at the protein level. Comparisons of the 5′ untranscribed regions showed that *Nbs2-Rdg2a* and *Nbs3-Rdg2a* were 93% identical in the 1,040 bp preceding the transcription start point (Figure S2), apart from a 347 bp insertion in *Nbs2-Rdg2a*, 145 bp upstream of the transcription start site. These findings suggest that the *Rdg2a* locus arose by gene duplication. A BLASTn search of the *Triticeae* Repeat Sequence (TREP) database (http://wheat.pw.usda.gov/ITMI/Repeats/) revealed 88% sequence identity between the insertion in the predicted promoter region of *Nbs2-Rdg2a* and members of the *Stowaway* class of miniature inverted transposable elements (MITEs). In contrast, *Nbs1-Rdg2a* showed only weak identity (51%) to the other two genes in the 700 bp preceding the transcription start (Figure S2). The three genes showed no significant similarity in the 3′-untranscribed regions.

To provide a comparison with a susceptible (*rdg2a*) genotype, we used gene-specific primers designed on the Thibaut *Nbs1-Rdg2a* and *Nbs2-Rdg2a* genes to obtain genomic sequences from cv. Mirco (GenBank accession numbers HM124453 and HM124454, respectively). Primers based on *Nbs1-Rdg2a* and *Nbs2-Rdg2a* genes yielded Mirco sequences with affiliation to the corresponding genes in Thibaut (Figure S2), suggesting that the amplified genes represented true alleles of the Thibaut genes. PCR markers based on insertion/deletions identified in the putative regulatory regions of the two genotypes (see below; Table S2), co-segregated with the *Rdg2a* locus in the high resolution mapping population (Figure S3, Methods S1), confirming that these two Mirco genes derive from the *rdg2a* locus.

Neither Mirco gene appears to be transcribed (see below), and this inactivity may be due to structural differences in the 5′ sequences (Figure 2B). Mirco *Nbs1-rdg2a* has a 436 bp insertion next to a putative TATA-box element, and a 854 bp insertion in the 5′ UTR with terminal inverted direct repeats of 138 bp. Neither insertion showed similarity to a known transposable element. Mirco *Nbs2-rdg2a* contained a 41 bp direct repeat just upstream of the transcription start site and lacked the MITE element present in the Thibaut gene (Figure 2B). Mirco *Nbs1-rdg2a* also contains frame shift mutations, resulting in a severely truncated ORF, whereas Mirco *Nbs2-rdg2a* contains an intact CC-NB-LRR ORF (Figure S4).

### *Nbs2-Rdg2a* expression, but not *Nbs1-Rdg2a*, is pathogen responsive

Semi-quantitative RT-PCR was performed using primer combinations specific for the *Nbs1-Rdg2a* and *Nbs2-Rdg2a* genes in either cv. Mirco or NIL3876-*Rdg2a* (Figure 2C; Table S3). In the susceptible cv. Mirco, neither gene showed detectable expression in embryos or leaves, even after increasing the number of PCR cycles and trying other primer combinations. In NIL3876-*Rdg2a*, expression of both genes was observed in uninoculated control embryos and in leaves of pathogen free plants. Some increase in transcript levels by 7 days after inoculation was evident for *Nbs2-Rdg2a* but not for *Nbs1-Rdg2a* (Figure 2C). Therefore, we performed quantitative RT-PCR in embryos of NIL3876-*Rdg2a* at five time points (7, 14, 18, 22 and 28 dai) (Fig. 2D). *Nbs2-Rdg2a* expression was significantly increased by inoculation at 7, 14, 18 dai (*P*<0.05, Methods S1) and was unresponsive by 22 dai, while *Nbs1-Rdg2a* expression was not appreciably altered by leaf stripe inoculation (Figure 2D).

### Identification of *Rdg2a*

**Figure 3:**
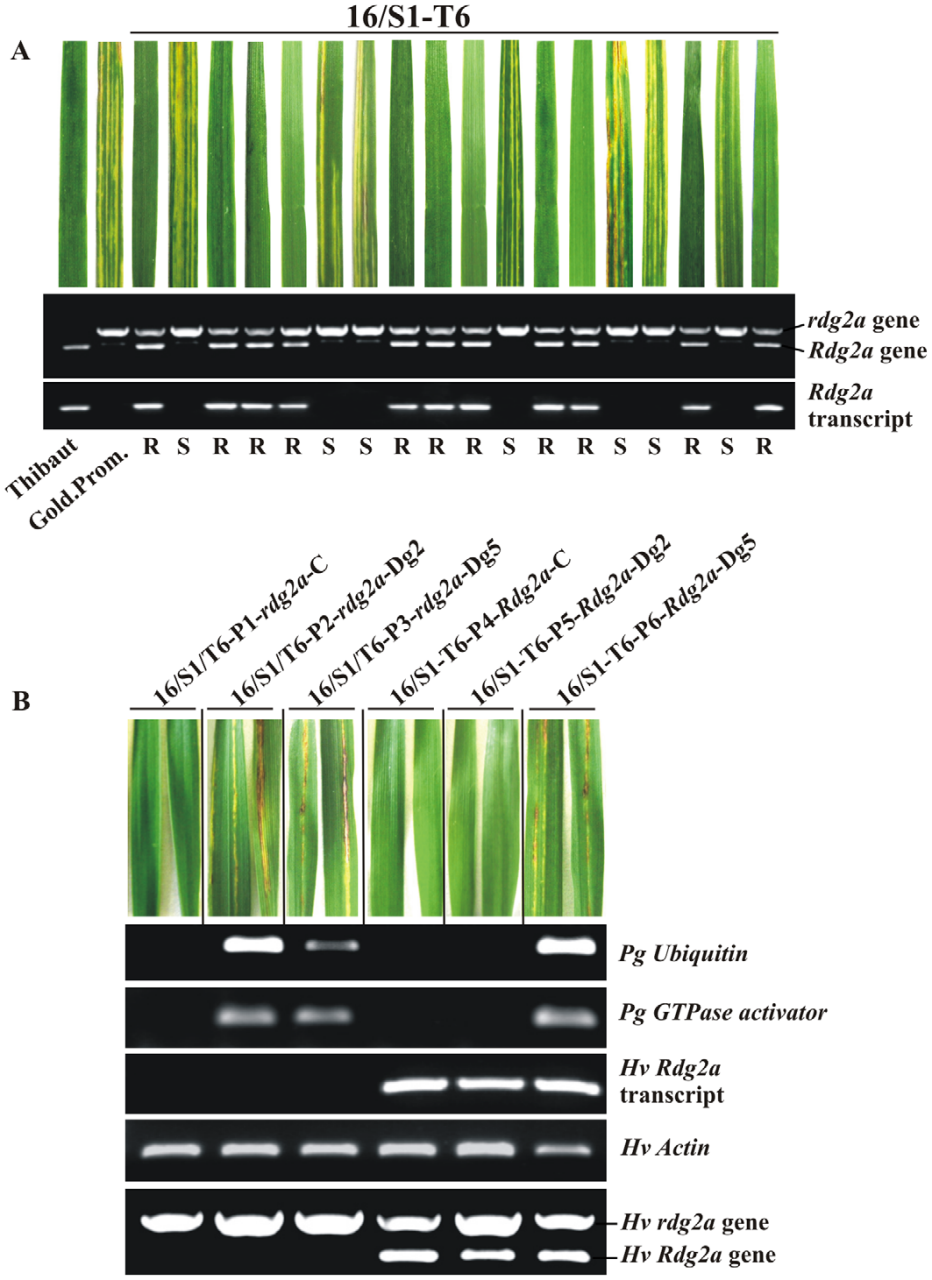
Analysis of T_1_ family 16/S1-T6 segregating for the *Nbs1-Rdg2a* transgene. (A) T_1_ seeds were inoculated with *P. graminea* isolate Dg2 and plants analyzed for disease symptoms in leaves (upper panel), an STS marker for *Rdg2a* (middle panel; upper band represents the *rdg2a* susceptibility allele from cv. Golden Promise while the lower band represents the *Rdg2a* transgene or endogenous gene), and *Rdg2a* transgene or endogenous gene expression by RT-PCR (lower panel). Resistance (R) or susceptibility (S) status of the plants is indicated underneath. The resistant cv. Thibaut and the susceptible cv. Golden Promise provide controls. (B) Leaves of six 16/S1-T6 T_1_ plants were analysed for expression of the fungal (*Pg*) *Ubiquitin* and *GTPase activator* genes and the barley (*Hv*) *Rdg2a* gene by RT-PCR. Seeds had been inoculated with Dg2 or Dg5 leaf stripe isolates or were non-inoculated (C). The barley *β*-*actin* gene was used as an internal control. Plant DNA was also tested for the presence of the transgene using the *Rdg2a* STS marker described in (A).

Genomic clones of the two *Rdg2a* candidates containing their native 5′ and 3′ regulatory sequences were used to transform the leaf stripe susceptible barley cv. Golden Promise. Ten randomly chosen T_0_ lines for each transgene were allowed to self-pollinate and the resulting T_1_ plants tested for resistance to isolates Dg2 and Dg5. This revealed that lines bearing the *Nbs1-Rdg2a* transgene were resistant to leaf stripe isolate Dg2 (Table 1). The overall escape rate of 5% among the null segregants was similar to the value observed in the susceptible control varieties (data not shown). Within T_1_ families, resistance to the same isolate co-segregated with the *Nbs1-Rdg2a* transgene and its expression (Figure 3A). These lines were susceptible to leaf stripe isolate Dg5, which is not recognised by *Rdg2a* (Table 1). T_1_ lines containing the *Nbs2-Rdg2a* transgene were fully susceptible to both the leaf stripe isolates (Table 1), although RT-PCR confirmed the transgene was expressed (data not shown).

**Table 1:** Analysis of transgenic plants.

		Isolate Dg2		Isolate Dg5	
Constructs/barley cvs.	Lines^a^	No. plants^a^	No. res. plants^b^	No. plants	No. res. plants
*Nbs1-Rdg2a*	1/S1-T6	19	19	15	0
	4/S1-T6	21	21	13	0
	7/S1-T6	24	24	11	0
	8/S1-T6	23	22	5	0
	16/S1-T6	19	19	12	0
	17/S1-T6	15	14	8	0
	19/S1-T6	7	7	9	0
	25/S1-T6	19	19	12	0
	31/S1-T6	13	13	5	0
	32/S1-T6	19	18	14	0
*Nbs2-Rdg2a*	41/S1-T7	23	1	17	0
	42/S1-T7	19	1	16	0
	46/S1-T7	16	0	9	0
	54/S1-T7	21	0	4	0
	56/S1-T7	17	1	5	0
	57/S1-T7	26	0	12	0
	60/S1-T7	20	2	16	0
	62/S1-T7	16	0	18	0
	64/S1-T7	17	0	7	0
	71/S1-T7	24	0	16	0
Thibaut (*Rdg2a*)		40^c^	38	6	0
NIL3876 (*Rdg2a*)		35	34	25	0
Mirco (*rdg2a*)		35	0	19	0
Golden Promise (*rdg2a*)		35	2	9	0
15/S1-T6 (empty vector)		36	1	15	0

a Made by transforming the susceptible barley cv. Golden Promise with the *Rdg2a* candidates *Nbs1-Rdg2a* or *Nbs2-Rdg2a*. Only those plants containing a transgene copy are included; null segregants are excluded.

b Number of transgenic T_1_ plants without leaf stripe symptoms. Data were pooled from three independent experiments each comprising 5 or more plants per line.

c Total number of plants tested as controls.

*Rdg2a* resistance terminates fungal growth in the embryo [5]. In the line 16/S1-T6 containing the *Nbs1-Rdg2a* transgene, plants challenged with the *P. graminea* isolate Dg2 showed no leaf stripe symptoms and there was no fungal mycelium in the leaves, indicated by undetectable transcripts of two fungal genes coding for *Ubiquitin* and *GTPase activator* (Figure 3B). In contrast, leaf stripe symptoms and fungal transcripts were observed in leaves of 16/S1-T6-*rdg2a* plants infected with Dg2 or Dg5 and 16/S1-T6-*Rdg2a* plants infected with Dg5 (Figure 3B).

As the *Nbs1-Rdg2a* gene could confer the same resistance specificity as *Rdg2a* in transgenic plants, we concluded that *Nbs1-Rdg2a* is *Rdg2a*.

### RDG2A protein structure

**Figure 4:**
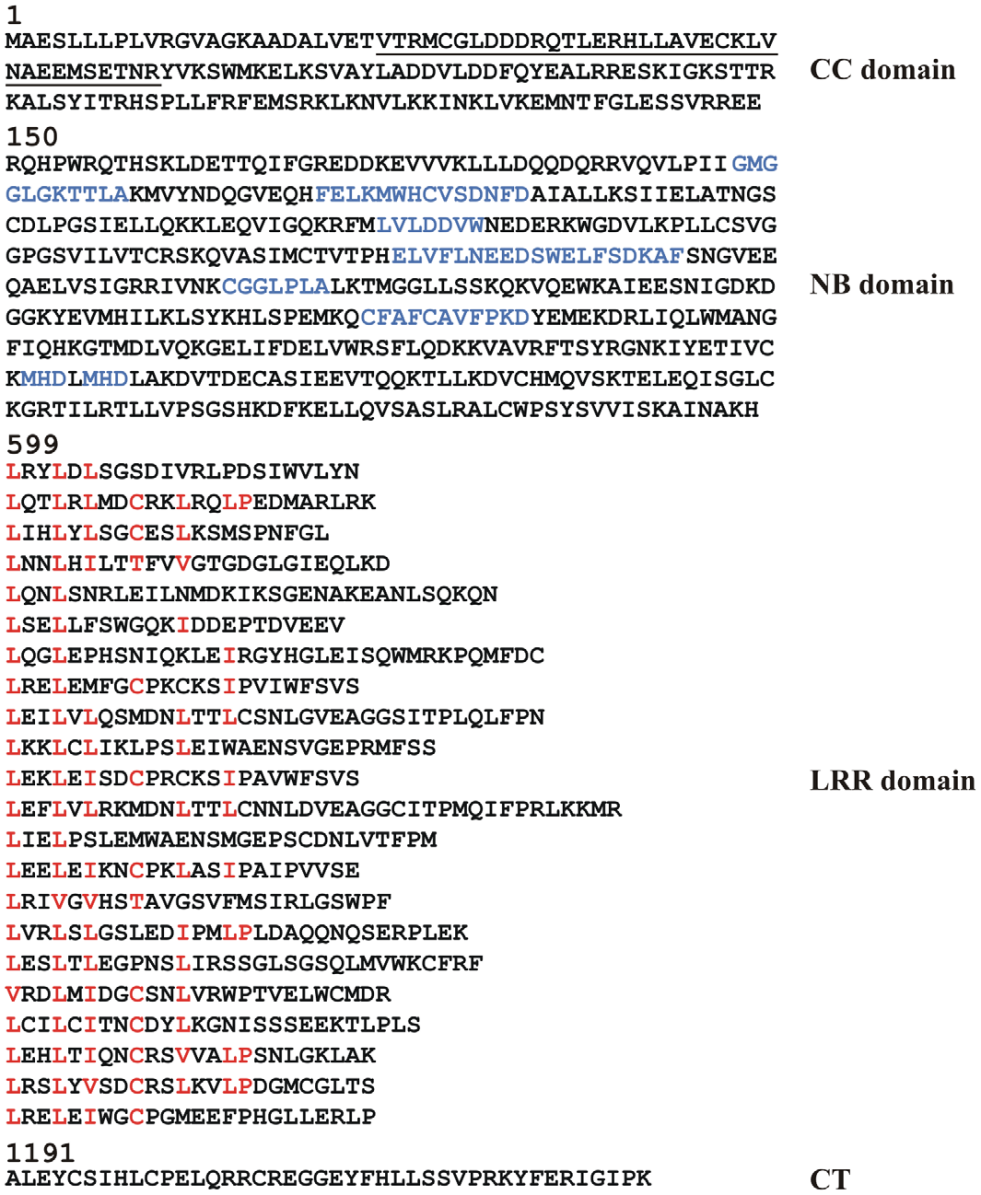
RDG2A protein sequence. The predicted coiled-coil (CC) domain is underlined. Motifs conserved in the NB region of NB-LRR proteins are in blue, and are (in order): P-loop, RNBS-A, Kinase 2, RNBS-C, GLPL, RNBS-D and MHD. Amino acids conforming to the cytoplasmic LRR consensus LxxLxLxx(C/N/T)xxLxxLxxLP are in red. CT denotes the RDG2A C-terminal region.

The predicted *Rdg2a* product of 1232 amino acids has an estimated molecular weight of 139.73 kDa. It contains all the conserved NB domain motifs of NB-LRR proteins defined by [18], [19], including the P-loop, RNBS-A, Kinase 2, RNBS-C, GLPL, RNBS-D and MHD domains, the latter of which is duplicated (Figure 4). A COILS analysis indicated the presence of a potential coiled-coil (CC) domain between amino acids 25 and 60, indicating that RDG2A belongs to the CC subset of NB-LRR resistance proteins [18]. The LRR region contains 22 imperfect repeats with a few repeats showing good agreement with the consensus motif LxxLxLxx(C/N/T)xxLxxLxxLP for cytoplasmic LRRs (Figure 4) [20].

**Figure 5:**
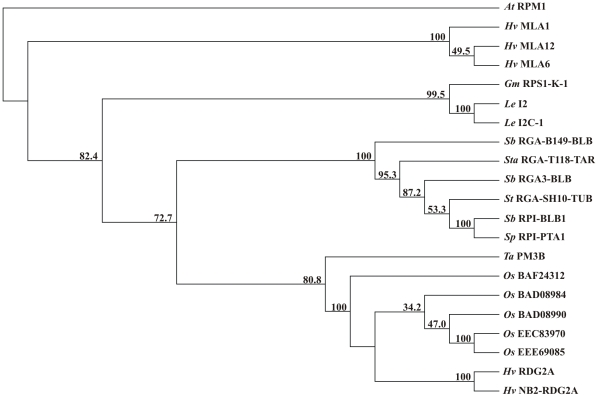
Neighbor-joining phylogenetic tree including RDG2A and similar resistance proteins and resistance gene analog products. Numbers on the branches indicate bootstrap percentages. Prefixes indicate species origin. The *A. thaliana* RPM1 protein (Q39214) was used as an outgroup. Shown are the rice (*Oryza sativa*) disease resistance-like proteins BAF24312, BAD08984, BAD08990, EEC83970 and EEE69085, the PM3 wheat powdery mildew resistance protein, products of the *S. bulbocastaneum* blight resistance gene *Rpi-blb1* and its paralogues *Rga3-blb*, and *Rpi-blb1*, predicted products of *RGA_B149.blb, RGA_T118-tar* (*S. tarijense*), *RGA_SH10-tub* (*S. tuberosum*) and *Rpi-pta1* (*S. papita*), the I2 and I2C-1 proteins encoded by the tomato (*Lycopersicon esculentum*) *I2* resistance locus to Fusarium wilt, the soybean (*Glycine max*) *Phytophthora* root rot resistance protein RPS-L-K-1, and the barley (*H. vulgare*) powdery mildew resistance proteins MLA1, MLA6 and MLA12.

Figure 5 illustrates similarities between RDG2A and the most similar sequences in the National Center for Biotechnology Information (NCBI) database. RDG2A was most similar (47–52%) over its whole length to five rice disease resistance-like proteins (accessions BAD08990, EEE69085, EEC83970, BAD0894, and BAF24312; Figure 5) encoded by genes clustered in a 2.97 Mbp region of rice chromosome 8 (nt. 25,872,241 to 28,845,527 of AP008214), which is not co-linear with the barley *Rdg2a* interval [7]. Of the known resistance proteins from barley, low levels (around 16%) of identity, restricted to the conserved motifs of the NB domain, were observed with the MLA1, MLA6 and MLA12 powdery mildew resistance proteins (Figure 5).

The RDG2A and NB2-RDG2A proteins are 75.3% identical, and differences include a deletion of three consecutive LRRs in NB2-RDG2A (Figure S5). Similarity is higher in the CC region than in the NB or LRR regions (Figure 4; 92.6 versus 73–74%), and the proportion of non-conservative amino acid substitutions is lower in the NB domain (75/104 = 72%) than in the LRR domain (57/71 = 80%). Similarly, the ratio of non-synonymous (*Ka*) to synonymous (*Ks*) nucleotide substitutions between *Rdg2a*, *Nbs2-Rdg2a* and *Nbs3-Rdg2a* (longest ORF) is 0.99, 2.13 and 2.63 for the CC, NB and LRR regions, respectively. Within the LRR domain, non-conservative substitutions are about twice as frequent in the β-strand/β-turn xxLxLxx motifs (solvent-exposed residues framed by aliphatic residues [20]) (Boxed, Figure S5) than elsewhere (25/133 = 18.8% versus 32/373 = 8.5%). These comparisons indicate that *Rdg2a* and its paralogues have been subjected to the highest level of diversifying selection in the LRR-coding region, consistent with the LRR domain being an important determinant of resistance specificity [21].

### Localization of RDG2A and NB2-RDG2A proteins to the nucleus and cytoplasm

**Figure 6:**
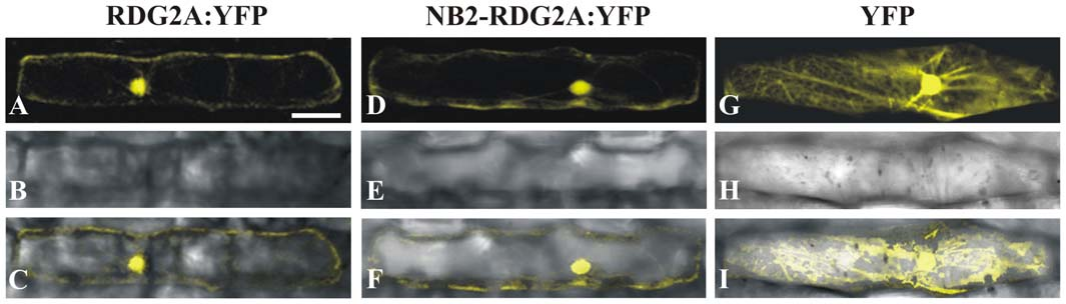
Sub-cellular localization of RDG2A and NB2-RDG2A proteins. Barley cv. Golden Promise epidermal cells were transiently transformed with constructs expressing RDG2A:YFP and NB2-RDG2A:YFP fusion proteins (A and D respectively), driven by the maize *polyubiquitin* gene promoter. A construct expressing YFP alone with the same promoter was used as control (G). Fluorescence signals were visualized using confocal laser scanning microscopy (A, D and G). Bright field images (B, E and H) and merged images (C, F and I) are shown. Scale bar represent 50 µm.

RDG2A does not have any predicted transmembrane domain or signal peptide sequence, suggesting a cytoplasmic location of the protein. To determine the subcellular location of the RDG2A and NB2-RDG2A proteins, we made 3′ fusions with the Yellow Fluorescent Protein (YFP) ORF and expressed the chimeric genes behind the maize *polyubiquitin* promoter. When either construct was transiently expressed in leaf epidermal cells of barley cv. Golden Promise, YFP fluorescence was clearly observed throughout the nucleus and also in the cytoplasmic strands (Figure 6). YFP alone has no nuclear localization signal but is smaller than the 40–60 kDa size exclusion limit of the nuclear pore complex [22]. Consistent with these characteristics, YFP expressed by itself was abundant in the cytoplasm and was also present in the nucleus (Figure 6).

### *Rdg2a* resistance does not involve hypersensitive cell death

**Figure 7:**
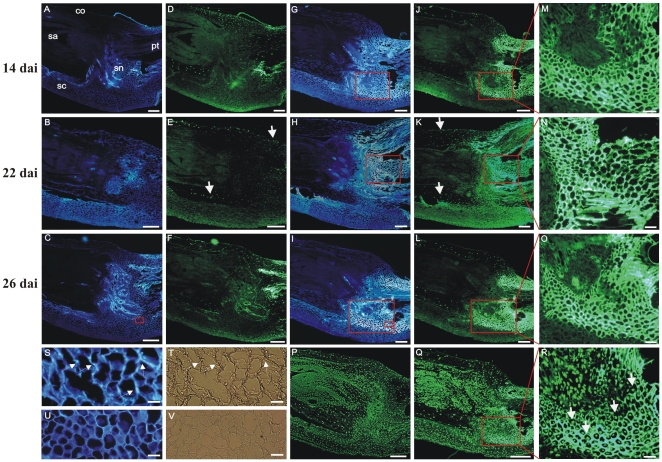
Histological analyses of NIL3876-*Rdg2a* barley embryos. (A) to (C) Sections of embryos grown under control conditions observed under UV excitation. (D) to (F) Sections in (A) to (C) subjected to TUNEL analysis. (G) to (I) Sections of embryos inoculated with leaf stripe isolate Dg2 and observed under UV excitation. (J) to (L) Sections in (G) to (I) subjected to TUNEL analysis; the bright green fluorescence at the level of scutellar node and provascular tissue is due to cell wall autofluorescence. (M) to (O) Magnified views of the boxed regions in (J) to (L) and (G) to (I). (S) and (T) Magnified views of the smaller box in (I) stained with calcofluor (S) or observed under bright field (T); arrows indicate the intercellularly growing *P. graminea* mycelium. (U) and (V) Magnified views of the small box in (C) stained with calcofluor (U) or observed under bright field (V). (P) and (Q) Respectively, sections of control and inoculated embryos at 26 dai, treated with DNase I and subjected to TUNEL analysis. (R) A magnified view of the region boxed in (Q). White arrows in Figure 7E, K and R indicate TUNEL positive nuclei. Scale bars represent 200 µM (A) to (L), 50 µM (M) to (O) and 25 µM (S) to (T). co  =  coleoptile, pt  =  provascular tissue, sa  =  shoot apex, sn  =  scutellar node.

*Rdg2a*-mediated resistance terminates fungal growth coincident with the appearance of cell wall-associated host-cell autofluorescence in tissues containing hyphae, mainly at the junction of the scutellum and scutellar node of the inoculated embryos [5]. Whole-cell autofluorescence is regarded as an indicator of HR in race-specific resistance of barley leaf epidermal cells to powdery mildew [23], [24] but was only occasionally (one or two cells per embryo section) observed in barley embryos expressing *Rdg2a* resistance. Nuclear DNA fragmentation is another PCD marker in plants [25]. However, while electrophoretic analysis of embryo DNA failed to detect it in association with *Rdg2a* resistance (data not shown), it is possible that DNA laddering went undetected due to the small proportion of pathogen-challenged cells that would have been present in the sample (cf. Figure 7). Therefore, we further tested for the presence of individual cells undergoing programmed death in the *Rdg2a* resistance response *in situ*, by using terminal deoxynucleotidyl transferase-mediated dUTP nick end labelling (TUNEL). This method enables detection of free 3′-OH groups created by DNA strand breaks that occur with programmed cell death. TUNEL was performed on serial sections of NIL3876-*Rdg2a* barley embryos (Figure 7). In non-inoculated embryos, no autofluorescence was observed under UV light (Figure 7A to C). In inoculated embryos, UV-autofluorescent tissues were observed at the scutellar node and provascular tissue at 14, 22 and 26 dai (Figure 7G, H and I respectively). Calcofluor staining and bright field observations revealed the presence of fungal mycelium in the tissues immediately adjacent to the autofluorescent regions (Figure 7S and T, respectively), indicating that autofluorescence was a genuine defence-associated response against leaf stripe. TUNEL revealed some nuclear DNA fragmentation (bright green fluorescent nuclei) in the coleoptile and in a few cells at the scutellar node of both non-inoculated (Figure 7D to F) and inoculated embryos (Figure 7J to L and M to O), however inoculation had no detectable effect on the frequency of these TUNEL signals. In the scutellar node and basal region of provascular tissue of the inoculated sample we observed, on average, 500 cells per section and time point of inoculation that were in contact with the fungus (on the basis of the calcofluor staining and bright field observations) and only one to two nuclei were positive to TUNEL staining. The same frequency of TUNEL positive nuclei was detected in the same regions of non inoculated embryos. Staining of the same sections with 4′,6-Diamidino-2-phenylindole (DAPI) dihydrochloride, verified the presence of intact nuclei in the autofluorescent regions (Figure S6). Following treatment of sections of control or inoculated embryos with DNaseI, TUNEL analysis stained all nuclei (Figure 7P to R), and no positive signals were observed in sections not treated with the deoxynucleotidyltransferase enzyme (data not shown), indicating that the TUNEL assay was working effectively.

## Discussion

### Evolution of the Rdg2a resistance locus

*Rdg2a* resides in a gene cluster, as does many other resistance genes. This organization can promote unequal recombination, which results in sequence exchange between paralogs and generation of recombinant genes with new resistance gene specificities, as well as expansion/contraction of gene copy number [26]. At the *Rdg2a* locus, paralogs appear to be the result of relatively recent gene duplication as indicated by the strong DNA sequence identity between the three NB-LRR genes that, in the case of *Nbs2-Rdg2a* and *Nbs3-Rdg2a*, extends into the 5′ untranscribed region (Figure S2). The unusual structure of *Nbs3-Rdg2a*, in which sequences encoding part of the NB and the LRR regions are duplicated, together with the deletion of the region containing three complete LRR units in NB2-RDG2A relative to RDG2A, provide further examples of variation at *Rdg2* locus generated by recombination.

Diversifying selection also contributes to sequence diversity at *R* gene loci [27]. However, this may only be the case for *R* genes that encode receptors that directly interact with pathogen effectors. *R* genes encoding proteins that act *via* an indirect guard mechanism, like RPM1 in Arabidopsis, are under conservative rather than divergent selection [28]–[30]. The functional alleles of these *R* genes would be conserved through evolution because they detect the presence of avirulence gene products that may not be able to mutate without a fitness penalty to the pathogen [31], [32]. Conversely, genes subjected to strong diversifying selection, like wheat *Pm3* or barley *Mla* alleles for race-specific powdery mildew resistance [33], [34], and Arabidopsis *RPP13* alleles for downy mildew resistance [35] in which sequence diversity is accompanied by functional diversity in pathogen recognition, are speculated to act through a model of direct interaction between *R* gene and *Av*r gene products [31], [36]. Our finding that *Rdg2a* was subjected to diversifying selection is consistent with a model in which the *R* gene co-evolves with a pathogen effector(s) gene, due to direct interaction of the two gene products. In this model, small conformational changes in the RDG2A protein restore the interaction with variant versions of the avirulence gene product, during an arms race between plant and pathogen. In such a model, genes for the leaf stripe avirulence products detected by RDG2A would also be under diversifying selection, similar to avirulence genes characterized in flax rust [37] and Arabidopsis downy mildew [38]. This view is also supported from the observation that in the only two leaf stripe susceptible barley genotypes analyzed to date, Mirco and Morex, sequences highly homologous to *Rdg2a* are present in syntenic position. In the barley cv. Morex, sequences sharing more than 93% of identity to *Rdg2a* were identified both in coding and non-coding regions and deletion(s) of intergenic regions and of members of the gene family (data not shown) are responsible of the rearrangements suggested by the Southern analysis (Figure S1).

Despite the fact that *Nbs2-Rdg2a* contains a complete open reading frame and is expressed in embryos, transgenic expression of *Nbs2-Rdg2a* failed to confer resistance to leaf stripe isolate Dg2. Analysis of near-isogenic lines indicated that the *Rdg2a* locus controls partial to strong resistance to at least 4 other isolates of the leaf stripe pathogen (Table S1). Whether the *Nbs2-Rdg2a* gene contributes any of these other resistance specificities is under investigation using the transgenic lines. The NB2-RDG2A and RDG2A proteins had multiple substitution differences in the NB and CC regions. However, there was only one (conservative) amino acid difference in the CC motif, and there were no differences in any of the motifs recognised as being conserved across the CC-NB-LRR class of resistance proteins (not shown). The LRR domains also showed a number of differences, including the deletion of three LRR units in NB2-RDG2A relative to RDG2A (Figure S5). Variation between R gene alleles or paralogues reported to abolish resistance function include both single amino acid substitutions [39], [40] and the absence or substitution of a section of the LRR domain encompassing one to several repeat units [41], [42]. Therefore, the substitutions or deletion within the LRR domain of NB2-RDG2A seem like plausible reasons for the absence of a resistance function for this protein. Transcript of *Nbs2-Rdg2a* was found to be 2 to 16 times less abundant than that of *Rdg2a*, depending on the time point and inoculation treatment (*P*<0.05, data not shown). Considering that transcript abundance correlates with resistance activity for the potato NB-LRR late blight resistance gene *RB* [43] and the rice receptor kinase-like bacterial blight resistance gene *Xa3* [44], lower expression of *Nbs2-Rdg2a* may contribute to its inactivity. This possibility will be explored by testing transgenic plants over-expressing *Nbs2-Rdg2a*. Complementation was not attempted using *Nbs3-Rdg2a*, which produces severely truncated proteins, and while a role of this gene in resistance would seem unlikely, we cannot yet rule it out. Insights into the functional consequences of this gene structure may be revealed by a current re-sequencing study, which aims to survey the *Rdg2a* locus haplotype variability and gene structure in other barley genotypes known to carry *Rdg2a* resistance specificities.

Strikingly, neither *Nbs1-rdg2a* nor *Nbs2-rdg2a* are transcribed in the susceptible cv. Mirco. Given the fitness cost of expressing some *R* genes [45], unnecessary *R* genes may become rapidly inactivated [46]. Rearrangements in the promoter region caused by insertion/deletion of transposable elements (Figure 2B) may explain the lack of expression of the Mirco genes. The alleles of *Nbs2-Rdg2a* are quite similar (93.1% identical), apart from the MITE insertion in the Thibaut allele. The PromH program for the prediction of plant promoters (http://www.softberry.ru/berry.phtml?group=programs&subgroup=promoter&topic=tssp, [47]) identified potential transcription factor binding sites, a TATA box, and a likely promoter within the MITE sequence (data not shown). It is therefore possible that sequences present in the MITE element contributed to the functionalization of this paralog, similar to the transcriptional activation of the rice blast resistance gene *Pit* by insertion of a *Renovator* retrotransposon into its 5′ region [48]. Although expression of NB-LRR *R* genes has only seldom found to be responsive to pathogen infection [49], [50], transcription of *Nbs2-Rdg2* was enhanced up to three fold by 14 days after inoculation by *P. graminea-Dg2* (Figure 2D), a time point when several defence-related genes are transcriptionally up-regulated in the *Rdg2a*-genotype [5]. It would be of interest to identify the regulatory sequences of *Nbs2-Rdg2a* involved in this pathogen responsiveness and determine whether these are located in the MITE insertion.

While the *Rdg2a* resistance allele from cv. Thibaut is used in breeding and still provides useful field resistance against leaf stripe disease, it is not effective against all isolates (Table S1) [51]. Therefore, identification of further alleles with different resistance specificity should have value, by broadening the range of resistance genes available to breeders and thus delaying the spread of virulent isolates. The cloning of *Rdg2a* should facilitate this task, by enabling sequencing and expression analysis of homologues from both wild and cultivated barley. Such an approach has led to the identification of functional *Pm3* alleles from both wild tetraploid and landraces of bread wheat [52], [53], allowing a significant expansion of the resistance gene repertoire available against powdery mildew in wheat.

### RDG2A localizes in the nucleus and cytoplasm, and confers resistance in the absence of programmed cell death

Fluorescence from transiently expressed RDG2A-YFP fusion protein was abundant in the nucleus and was also present in the cytoplasm, suggesting that resistance functions of RDG2A might relate to one or both of these locations. A nuclear activity of a NB-LRR protein mediated by a WRKY transcription factor was previously demonstrated for the powdery mildew resistance protein MLA10 in barley [54]. MLA10 interacts with WRKY1 in the nucleus in the presence of the *Blumeria graminis* effector AVR_A10_, leading to a de-repression of basal defence mechanisms and effective immunity [54]. We previously observed that a *WRKY1* allele (designated *WRKY38* in [55]), is up-regulated upon *P. graminea*-Dg2 infection [5]. Therefore, it may be worth testing if RDG2A interacts with WRKY38 and whether this interaction is required for the resistance response. It should however be noted that we determined subcellular localization in leaves of uninfected plants, and that the location of the resistance protein might differ in barley embryos inoculated with *P. graminea*. Irrespective of this, the intracellular localization of RDG2A would imply that the recognition of avirulence gene products occurs inside the host cell and that the leaf stripe *Avr* gene products are transported across the plasma membrane during the infection. This is notable given that the leaf stripe fungus only grows between cells [3], [5], suggesting that there must be a mechanism for delivery of the avirulence protein into the host cell. In contrast, several characterized *Avr* gene products of *Cladosporium fulvum*, a pathogenic fungus of tomato that shares with *P. graminea* an intercellular mode of pathogenesis, are in each case recognized by membrane-anchored resistance proteins containing extracellular LRRs [56].

While HR is a common component of resistance gene-mediated defence and often used as surrogate for resistance protein activity, there are a few known cases of NB-LRR genes conferring resistance without HR, at least based on the failure to observe macroscopically visible host cell death. For example, the barley *Mla1* powdery mildew resistance gene can trigger an immune response without macroscopically visible HR [57] although the *Mla12* allele exhibits clearly a necrotic reaction [58]. It has been proposed that the absence of HR associated with resistance to potato virus x governed by the *Rx* gene in potato is because the resistance mechanism is so rapid, preventing accumulation of the avirulence factor to levels that would otherwise trigger a more extensive host response [59]. Similarly, naturally occurring alleles of Arabidopsis *RPS4* or *RPS6* confer bacterial resistance without development of an HR [60]. In the current study, TUNEL positive nuclei were observed in the scutellum and in the coleoptile both in control and inoculated embryos. However, inoculation did not increase the frequency or distribution of these signals. Therefore, these observations most likely reflect cell death that normally occurs with development, as previously observed in barley germinating seeds and in the corresponding cells of the scutellum and coleoptile of maize embryos [61], [62]. In HR of barley epidermal cells against the biotrophic powdery mildew fungal pathogen governed by the *Mla12* resistance gene, autofluorescence and accumulation of phenolic compounds is observed throughout the whole host cell [23], [24]. Autofluorescence at the junction of the scutellum and scutellar node regions was observed in the resistance response to leaf stripe, but was essentially confined to the cell walls and only occasionally observed throughout a whole cell (this study, [5]). No necrotic tissues or cell collapse was observed under bright views of the embryo regions showing autofluorescence (data not shown), further indicating that hypersensitive cell death did not occur. One could speculate that an HR-associated resistance response would be too damaging to the embryo, and therefore unviable in an evolutionary sense. HR deprives obligate biotrophic pathogens of living host cells required for successful colonization, but may be favourable to the hemibiotrophic leaf stripe pathogen, which obtains nutrients at latter stages of colonization by means of hydrolytic degradation of host cell walls. *Rdg2a* resistance terminates *P. graminea* mycelium growth at the scutellar node and basal regions of provascular tissue of the barley embryos, and is associated with the accumulation of phenolic compounds in the cell walls of the invaded host tissues. These phenolic compounds are the likely source of the cell wall localized autofluorescence. Also pathogen-induced up-regulation of several genes related to cell wall modification was observed in the resistant NIL but not in the susceptible one [5]. We therefore propose that inducible secretory immune responses, leading to physical and chemical barriers to infection in the cell walls and intercellular spaces of the barley embryo tissues, represent mechanisms by which the CC-NB-LRR-encoding *Rdg2a* gene mediates resistance to leaf stripe.

## Materials and Methods

### Plant and fungal materials

Genetic mapping was performed using 93 F_2_ recombinants for the 3.47-cM *Rdg2a* marker interval ABG704-ScOPQ9, previously selected from an F_2_ population of 1,400 plants made from a cross between barley cvs. Thibaut (resistant, *Rdg2a*) and Mirco (susceptible, *rdg2a*) [7]. NIL3876- *Rdg2a* contains the *Rdg2a* gene from Thibaut backcrossed into the genetic background of Mirco [10]. Barley cv. Morex was used for Southern-blot experiments while the susceptible variety Golden Promise was used for transformation tests. The leaf stripe (*P. graminea*) isolates Dg2 (incompatible on *Rdg2a*) and Dg5 (compatible on *Rdg2a*) were used in our study. The Dg2 isolate is the most virulent isolate in a previously described collection of monoconidial isolates [51]. The *P. graminea* isolates were grown on PDA (Liofilchem, Italy), in Petri dishes at 20°C for 10 days in the dark. Seeds were surface-sterilized in 70% ethanol for 30 s and then in 5% sodium hypochlorite for 15 min prior to inoculation using the ‘sandwich’ technique [63].

### Generation of transgenic barley lines

Genomic DNA fragments of about 6 kb were used in transformation experiments, and for *Nbs1-Rdg2a* and *Nbs2-Rdg2a*, included 1196 or 985 bp of 5′ untranscribed sequence, and 556 or 658 bp of 3′ untranscribed sequence, respectively. These were PCR amplified using primer sequences provided in Table S4 and Phusion HF Taq DNA polymerase (New England Biolabs), from cosmid 95-9-3 (*Nbs1*-*Rdg2a*) and cosmid 17-1-1 (*Nbs2-Rdg2a*), subcloned in pDONR201 (Invitrogen) and then transferred to the Gateway (Invitrogen) compatible version of the *Agrobacterium* binary vector pWBVec8 [64]. Inserts were confirmed as having the same sequence as the cosmid clones. Transgenic barley plants were generated by co-cultivation of *Agrobacterium tumefaciens* with immature barley embryos of cv. Golden Promise, as described by [57]. Transgenes were detected by PCR with the gene-specific primer pair Nbs1_25 and Nbs1_26 (Table S3) that amplified a 387 bp fragment in Thibaut and a 500 bp fragment in Golden Promise. Transgene copy number for *Nbs1-Rdg2a* was evaluated by Southern hybridization analysis of genomic DNAs digested with *Eco*RI and *Kpn*I, which respectively have one and two restriction sites in the *Rdg2a* genomic sequence used for transformation. This identified one single copy integration for all the lines but one multiple copy integration for line 8/S1 (data not shown).

### Subcellular localization of RDG2A and NB2-RDG2A

To generate the YFP fusion constructs, the coding sequences of *Nbs1-Rdg2a* and *Nbs2-Rdg2a* were firstly amplified from the aforementioned pDONR201 entry clones using 15 ng of plasmid DNA with Phusion HF *Taq* DNA polymerase (New England Biolabs) according to manufacturer's instructions, and the products transferred into a Gateway destination vector (pUbi-Gateway-eYFP) previously used in barley transient expression studies [65]. The constructs contain the *Nbs1-Rdg2a* and *Nbs2-Rdg2a* ORFs 3′-fused with the *YFP* ORF, behind the maize ubiquitin promoter. Transient gene expression in barley epidermal cells was performed by particle bombardment as previously described by [66]. Fluorescence imaging was performed using a TCS SP2 AOBS confocal laser-scanning microscope (Leica), with the 514-nm Ar/Kr- ion laser line used to excite YFP, and 525–580 nm used for image collection. Images were collected and processed using the software LCS (Leica). Reference emission spectra of YFP was used to discriminate genuine YFP emission fluorescence from nonspecific background fluorescence.

### Histology

Sections of inoculated (14, 22 and 26 dai) and control embryos were fixed in freshly prepared 4% p-formaldehyde in phosphate-buffered saline (PBS) pH = 7 (130 mM NaCl, 7 mM Na2HPO4, 3 mM NaH2PO4) for 12 hours and then stored in 70% ethanol at 4°C until use. The terminal deoxynucleotidil transferase-mediated dUTP nick end labelling (TUNEL) assay was performed according to the manufacturer's instructions (Roche Diagnostics, Mannheim, Germany), and nuclei were stained by incubating in 1 mM 4′,6-Diamidino-2-phenylindole (DAPI) for 20 min. For TUNEL analysis, three independent replicate experiments were performed. Per experiment, six embryos (five sections for each embryo) were observed per time point and inoculation status. For TUNEL assay, a negative control was provided by omitting terminal deoxynucleotidyl transferase enzyme, and a positive control was provided by treating samples with DNase1. For calcofluor staining, sections were incubated in 0.01% calcofluor in PBS pH 7 for 30 min. Samples were observed with an Olympus BX51 microscope with the settings (a) excitation at 451–490 nm and emission at 491–540 for fluorescein, or (b) excitation at 335–380 nm and emission at >420 nm for autofluorescence, DAPI and calcofluor staining. Images were recorded using an Olympus DP50 microscope digital camera system.

## Supporting Information

Table S1 *Rdg2a* resistance spectrum.
(0.03 MB DOC)

Table S2 Details of genetic markers.
(0.04 MB DOC)

Table S3 Sequences of PCR primer sets and annealing temperatures used in the expression analyses.
(0.04 MB DOC)

Table S4 PCR primers used to generate constructs for barley transformation.
(0.04 MB DOC)

Figure S1 Southern blot analysis of *Rdg2a* candidates. BamHI-digested barley genomic DNA was hybridised with probes derived from the LRR region of the NB-LRR genes.
(8.07 MB TIF)

Figure S2 DNA sequence homologies between paralogs and alleles at the *Rdg2a* leaf stripe resistance locus. Diagrams above define the domains compared. Percent identities were determined once major insertions/deletion differences had been removed.
(9.88 MB TIF)

Figure S3 Demonstration that the sequenced Mirco *Nbs1-rdg2a* and *Nbs2-rdg2a* genes represent alleles of the respective Thibaut genes. Markers Nbs1-14-19 and Nbs2-6-29 developed using insertion/deletion polymorphisms in the putative regulatory regions (A) co-segregated with the *Rdg2a* locus in 12 rare recombinants for the *Rdg2a* region that had been identified in the high resolution mapping population (B). Recombination points are illustrated below (C).
(9.85 MB TIF)

Figure S4 Predicted ORF and putative protein domains encoded from the Mirco genes *Nbs1-rdg2a* and *Nbs2-rdg2a*.
(9.84 MB TIF)

Figure S5 Alignment of the deduced LRR domain sequences of RDG2A and NB2-RDG2A. Substitution differences are boxed; those in grey and green represent conservative and non-conservative substitutions (as defined by ClustalW), respectively. The regions of the LRRs that correspond to the β-strand/β-turn motif xxLxLxx are framed and the leucine (or other aliphatic) residues that form the structural backbone of the LRR units in RDG2A are in red.
(9.58 MB TIF)

Figure S6 DAPI staining of embryo sections analyzed for autofluorescence and by TUNEL in Figure 7. DAPI staining of nuclei was performed for embryo sections of Figure 7 A and D (A), B and E (B), C and F (C), G and J (D), H and K (E), I and L (F). Scale bars represent 200 µM.
(9.88 MB TIF)

Methods S1 Supplementary text for Materials and Methods.
(0.06 MB DOC)
