# Supplementary material for: The CC-NB-LRR-Type Rdg2a Resistance Gene Confers Immunity to the Seed-Borne Barley Leaf Stripe Pathogen in the Absence of Hypersensitive Cell Death
Source: PLoS One. 2010 Sep 10;5(9):e12599. doi: 10.1371/journal.pone.0012599 (PMC2937021; doi:10.1371/journal.pone.0012599)
Supplement: Table S1 — Rdg2a resistance spectrum. (0.03 MB DOC) [file pone.0012599.s001.doc]

**Table S1.** *Rdg2a* resistance spectrum

|  | **Leaf stripe isolate** | | | | | | | |
| --- | --- | --- | --- | --- | --- | --- | --- | --- |
| **Barley genotype** | **Dg1** | **Dg2** | **Dg4** | **Dg5** | **Dg10** | **Dg12** | **Dg19** | **Dg23** |
| NIL3876-*Rdg2a* | 91a | 100 | 32 | 5 | 60 | 92 | 100 | 40 |
| Mirco-*rdg2a* | 66 | 5 | 20 | 5 | 11 | 41 | 44 | 3 |

aPercentage of plants without leaf stripe symptoms. Data were pooled from three independent experiments each comprising 15 plants per line.
