# Supplementary material for: The CC-NB-LRR-Type Rdg2a Resistance Gene Confers Immunity to the Seed-Borne Barley Leaf Stripe Pathogen in the Absence of Hypersensitive Cell Death
Source: PLoS One. 2010 Sep 10;5(9):e12599. doi: 10.1371/journal.pone.0012599 (PMC2937021; doi:10.1371/journal.pone.0012599)
Supplement: Table S2 — Details of genetic markers. (0.04 MB DOC) [file pone.0012599.s002.doc]

**Table S2.** Details of genetic markers

| **Marker**  **Name** | **Primers** | **Annealing** | **Exp. Size undigested** | **Restriction enzyme** | **Type** |
| --- | --- | --- | --- | --- | --- |
| 851-1F-1R | 851-F 5’-ATGACAAGCTACACGGCACCAACTACGCC  851-R 5’-GACACTCGGTGAAATGCAGCGCATC | 65°C | 1100 | *Acy*I | CAPS |
| 146-1F-1R | 146G201F 5’-CAGGACAATGTCAATGTCAGTCAT  146G20-1R 5’-CCTATGTTTGTCACCTTGGTTT | 60°C | 300 | *Bst*1107I | CAPS |
| 146-4F-3R | 146G204F 5’-GGTCTCTTCTCGCGAGCCTCACTCCAA  146G203R 5’-ATGCAGCCGCGGGTGTAGTTGGAT | 60°C | 295 | *Dde*I | CAPS |
| 146-6F-5R | 146 6F 5’-GCAAAGAGATGCTCATGCCTAAAGACCC  146 5R 5’-GGGCAGGGAGTAGTAGACTGAACATCAT | 60°C | 260 | *Taq*I | CAPS |
| 630-39 1-2 | 630-39 1 5’-CCAAATCAGACACGGGAGTAGTATGCAG  630-39 2 5’-AGCTTTGCGCCCGGATCTGATC | 65°C | 240 | *Taq*I | CAPS |
| 146-30 3-4 | 146-30 3 5’-CTCTGTTGGTACAACTCGAAG  146-30 4 5’-CTAGACGAGGCCCAACTCTGTC | 65°C | 242 | *Bsr*I | CAPS |
| 146-9 5-6 | 146-9 5 5’-TATGCAAAGGCAGAACATACCTCCGC  146-9 6 5’-GCAACTCCTTGTGTGATCGCTTGGAAGCT | 60°C | 108 | *Hind*III | dCAPS |
| 608-32 3-4 | 608-32 3 5’-GATGGAGACGATTTCTGAAAGAGGATGAGACGTCT  608-32 4 5’-CCCGATCAATAACCGTCGGTGTCTCTATTACC | 60°C | 116 | *Bsm*AI | dCAPS |
| 146-60 1-2 | 146-60 1 5’-GGCTCCTGGCCATTTCTCGTCA  146-60 2 5’-CCATCAATCGTCAGATCTGTCACG | 60°C | 221 | *Nla*III | CAPS |
| 608-16 3-4 | 608-16 3 5’-CTAGGCCTCCGCCACCGTCCCCATCGACGGCTC  608-16 4 5’-CCGAGTCTCCGTTATACGAAAC | 60°C | 90 | *Bsp*LI | dCAPS |
| 608-26 1-2 | 608-26 1 5’-CCTTTCCTAGGAGGTGGAGCAAGC  608-26 2 5’-TGACAAGTGGAAGGAAGATGACGGG | 65°C | 236 | *Nco*I or *Hpa*II | CAPS |
| 146-40 RFLP NBS | 146-40-F 5’- CCTTCAAGCGCATAAAACAT  146-40-R 5’- CTCTAATATTCGCAATGTCG |  | 695 | *Eco*RI | RFLP |
| Nbs1 14+19 | Nbs1-14 5’- TACTTGGTTTGGAGCTAGGAGACG  Nbs1-19 5’- GGTACCATCGATTCATGACGTTAGCAT | 65°C |  |  | STS |
| Nbs2 6+29 | Nbs2-6 5’-GCAGAAGAATGCCTACAAAACCCTGAGTCC  Nbs2-29 5’- CAAGGTAAGGATTGAGGAGAGC | 65°C |  |  | STS |
