# Supplementary material for: The CC-NB-LRR-Type Rdg2a Resistance Gene Confers Immunity to the Seed-Borne Barley Leaf Stripe Pathogen in the Absence of Hypersensitive Cell Death
Source: PLoS One. 2010 Sep 10;5(9):e12599. doi: 10.1371/journal.pone.0012599 (PMC2937021; doi:10.1371/journal.pone.0012599)
Supplement: Table S3 — Sequences of PCR primer sets and annealing temperatures used in the expression analyses. (0.04 MB DOC) [file pone.0012599.s003.doc]

**Table S3.** Sequences of PCR primer sets and annealing temperatures used in the RT-PCR and qRT-PCR expression analyses.

| **Gene** | **Primer combination** | **Sequence** | **Annealing temperature** |
| --- | --- | --- | --- |
| Nbs1-Rdg2a | Nbs1_25  Nbs1_26 | GATGAGCCTACAGATGTGGAAGAAGTGC | 60°C |
|  |  | GCTAAACATCCGAGGCTCTCCTACACTA |  |
|  | Nbs1_27  Nbs1_28 | TTTCATCATCCGAGGAGAAAACCCTTCCGC | 60°C |
|  |  | GCCGATTCACTTTGGGATGCCTATTCTCTC |  |
| Nbs2-Rdg2a | Nbs2_2  Nbs2_3 | GGAGATTCAGGTCTGCCGCAGAGTG | 60°C |
|  |  | TTTGTTATCTCCTTCAGAATCATGGGAG |  |
|  | Nbs2_4  Nbs2_5 | GAAGCACTTCTTCCACATTACAGGCC | 60°C |
|  |  | CGGGCAGCCACGTATGCTAAAGG |  |
| Nbs1-rdg2a | Nbs1_1m  Nbs1_2m | CCAACAGCTTGATCAGAAGCTCCGA | 60°C |
|  |  | ACCAGACTTCGGCAGCCCCAAATCA |  |
|  | Nbs1_3m  Nbs1_4m | TGATTTGGGGCTGCCGAAGTCTGGT | 60°C |
|  |  | CGATTTACTTCACAGCTACTATTCT |  |
| Nbs2-rdg2a | Nbs2_5m  Nbs2_6m | TGATTCTGAAAAGGCCCAACAGTT | 60°C |
|  |  | TTAGGCTCATGCAGTCCTCCACCCA |  |
|  | Nbs2_7m  Nbs2_5 | TGGGTGGAGGACTGCATGAGCCTAA | 60°C |
|  |  | CGGGCAGCCACGTATGCTAAAGG |  |
| β-Actin | Actin_Forward  Actin_Reverse | ATGTGGCCATCCAGGCAGTGCTTT | 60°C |
|  |  | TGGTCTCATGGATTCCAGCAGCTTCC |  |

Primers for fungal genes *Ubiquitin* and *GTPase activator* are reported in Haegi et al. 2008
