# Supplementary material for: The CC-NB-LRR-Type Rdg2a Resistance Gene Confers Immunity to the Seed-Borne Barley Leaf Stripe Pathogen in the Absence of Hypersensitive Cell Death
Source: PLoS One. 2010 Sep 10;5(9):e12599. doi: 10.1371/journal.pone.0012599 (PMC2937021; doi:10.1371/journal.pone.0012599)
Supplement: Table S4 — PCR primers used to generate constructs for barley transformation. (0.04 MB DOC) [file pone.0012599.s004.doc]

**Table S4.** PCR primers used to generate constructs for barley transformation.

| **Gene** | **Primer combination** | **Sequence** | **nt from ATG (5’ primer) /from STOP codon (3’ primer)** | **Annealing temperature** |
| --- | --- | --- | --- | --- |
| Rdg2a- Nbs1 | Nbs1_ aTrasf_F1  Nbs1_Transf_R1 | TCCTGGCCGGTTGATGGCTTT | -1472 | 60°C |
|  |  | CGGGAGTGCATTGAAGAAGGGTTG | +658 |  |
|  | Nbs1_bFusion_F1  Nbs1_Fusion_R2 | cACatggcagagtcactccttctccctctag | 0 | 55° C |
|  |  | CTTTGGGATGCCTATTCTCTCAAAGTATTTAC | -3 |  |
| Rdg2a- Nbs2 | Nbs2_Trasf_F1^2^  Nbs2_Trasf_R1 | TGTCTGCACAGGAAGGATTCTGGTCA | -1313 | 60°C |
|  |  | TGGCGTGCGTTTCGTTGTGC | +806 |  |
|  | Nbs2_Fusion_F1^2^  Nbs2_Fusion_R1 | ACatggcagagtcactccttctccctcta | 0 | 55° C |
|  |  | ACAGTCTTTACGTGGGACAGAGGAGAGC | -3 |  |

aTrasf primers were used to generate constructs for *Agrobacterium*-mediated stable transformation

bFusion primers were used to generate constructs in which the YFP coding sequence was fused in frame to the C terminal region of the *Rdg2a* candidate

cThe AC bases underlined in the fusion primers were introduced to maintain the frame within the gateway cassette.
