# Supplementary figures and images for: The CC-NB-LRR-Type Rdg2a Resistance Gene Confers Immunity to the Seed-Borne Barley Leaf Stripe Pathogen in the Absence of Hypersensitive Cell Death
Source: PLoS One. 2010 Sep 10;5(9):e12599. doi: 10.1371/journal.pone.0012599 (PMC2937021; doi:10.1371/journal.pone.0012599)

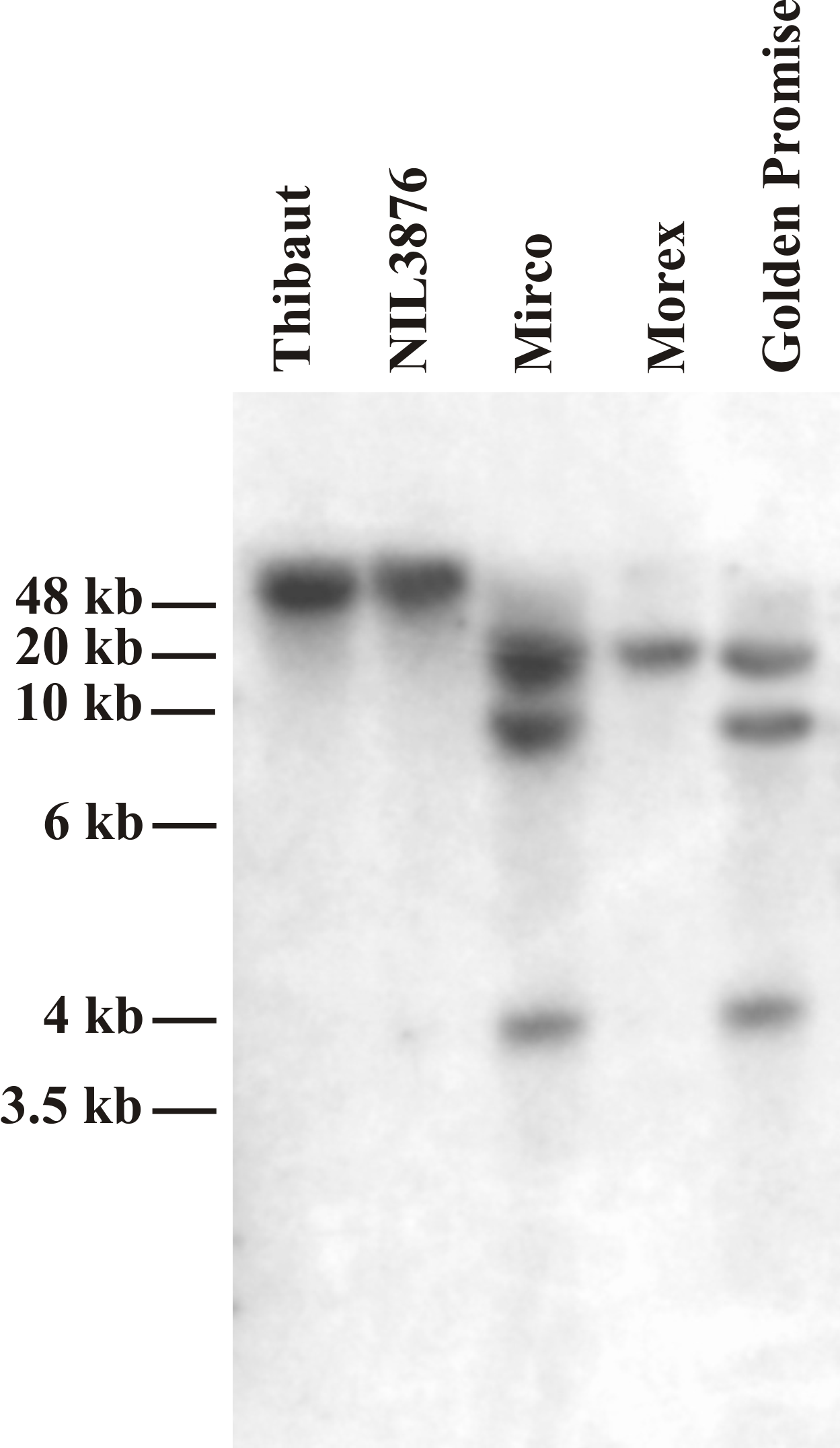

Supplement: Figure S1 — Southern blot analysis of Rdg2a candidates. BamHI-digested barley genomic DNA was hybridised with probes derived from the LRR region of the NB-LRR genes. (8.07 MB TIF) [file pone.0012599.s005.tif]

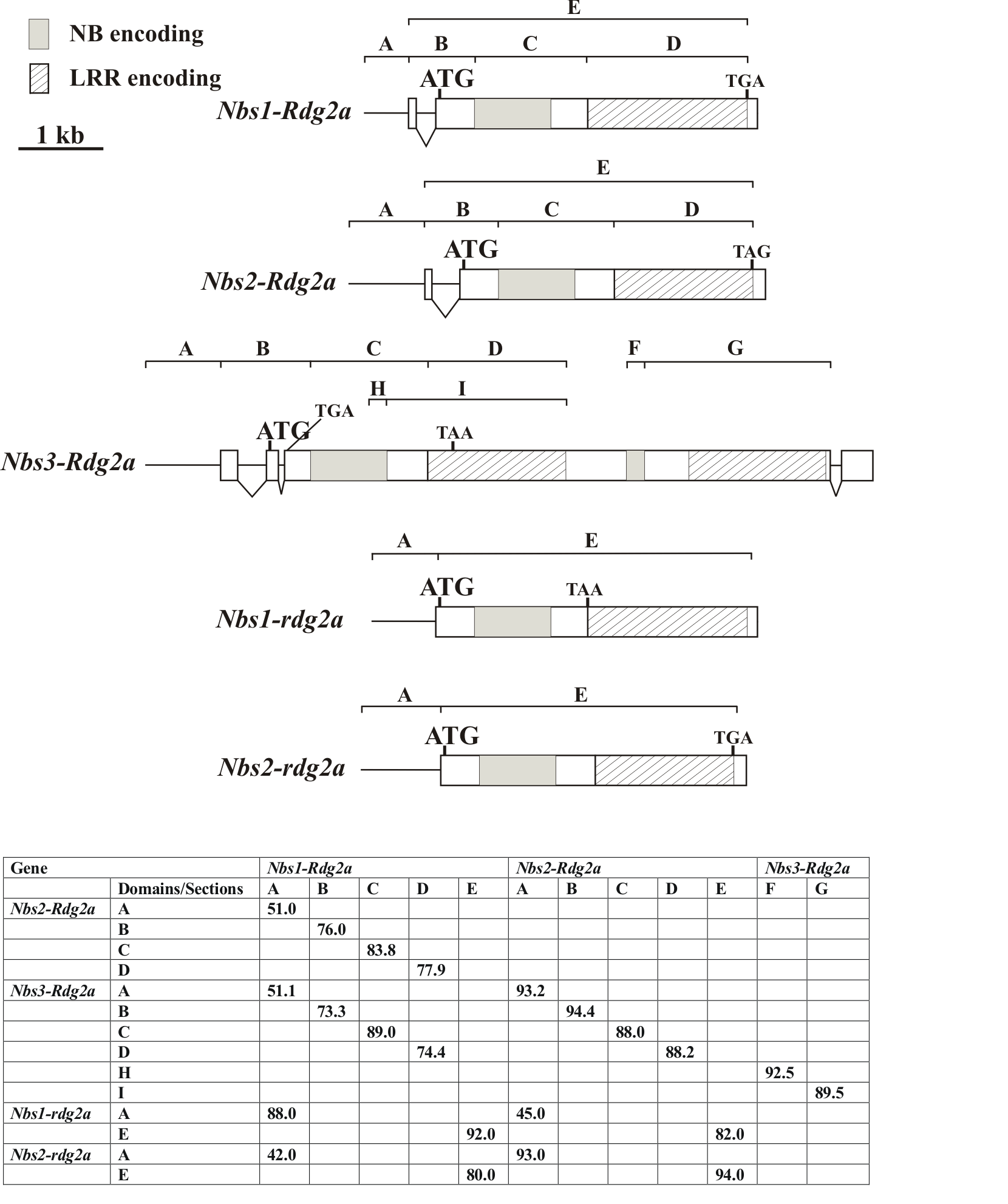

Supplement: Figure S2 — DNA sequence homologies between paralogs and alleles at the Rdg2a leaf stripe resistance locus. Diagrams above define the domains compared. Percent identities were determined once major insertions/deletion differences had been removed. (9.88 MB TIF) [file pone.0012599.s006.tif]

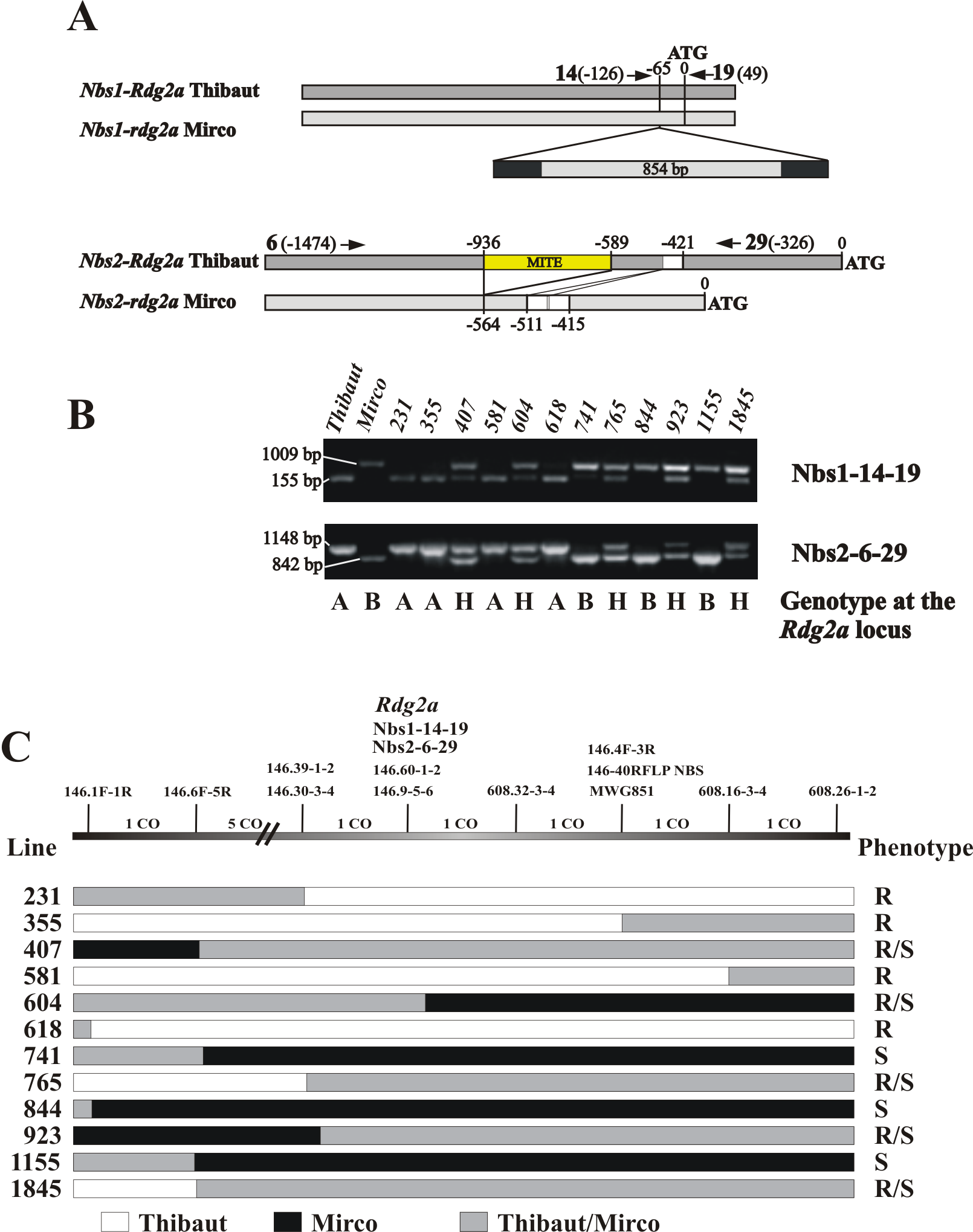

Supplement: Figure S3 — Demonstration that the sequenced Mirco Nbs1-rdg2a and Nbs2-rdg2a genes represent alleles of the respective Thibaut genes. Markers Nbs1-14-19 and Nbs2-6-29 developed using insertion/deletion polymorphisms in the putative regulatory regions (A) co-segregated with the Rdg2a locus in 12 rare recombinants for the Rdg2a region that had been identified in the high resolution mapping population (B). Recombination points are illustrated below (C). (9.85 MB TIF) [file pone.0012599.s007.tif]

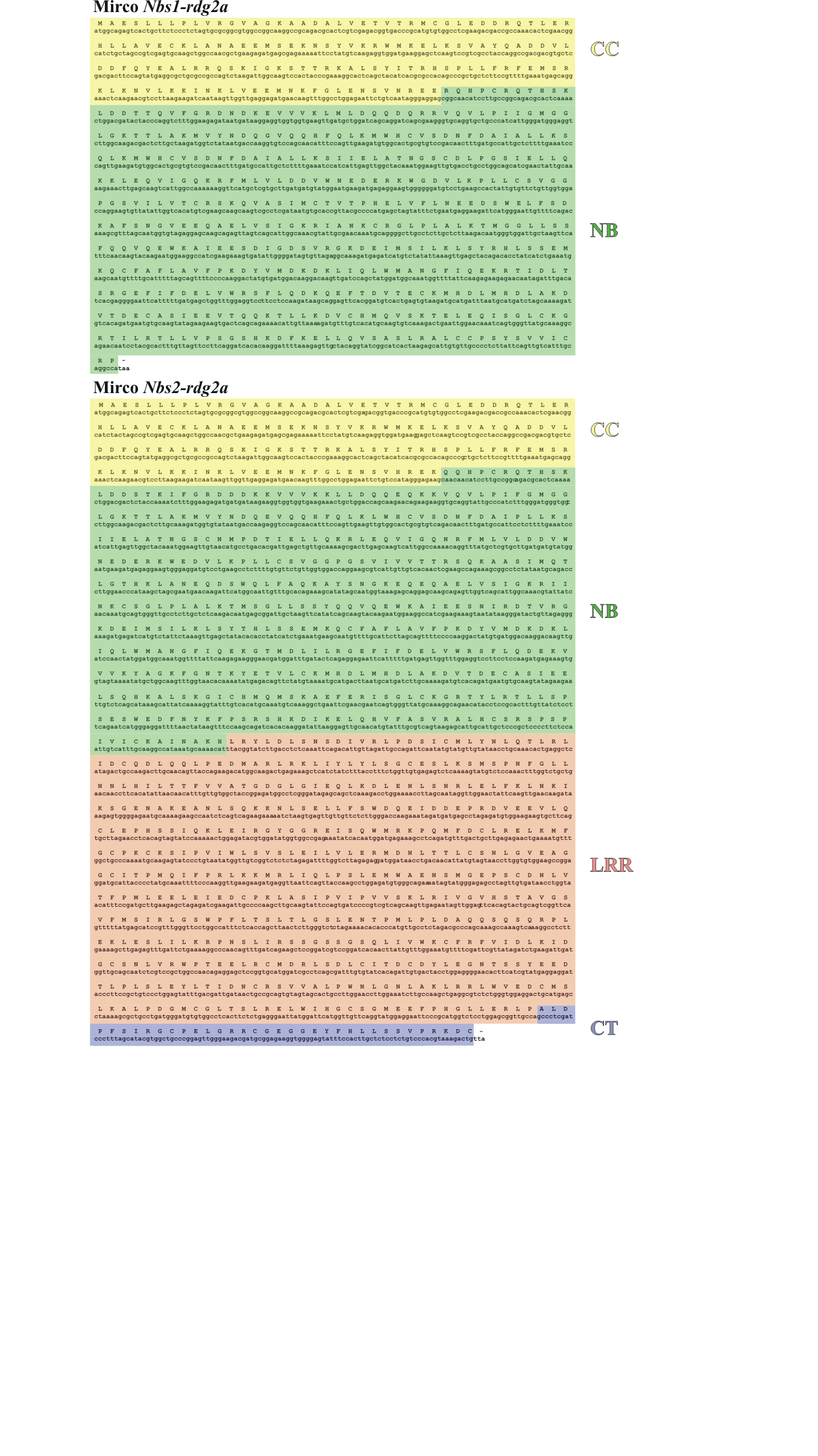

Supplement: Figure S4 — Predicted ORF and putative protein domains encoded from the Mirco genes Nbs1-rdg2a and Nbs2-rdg2a. (9.84 MB TIF) [file pone.0012599.s008.tif]

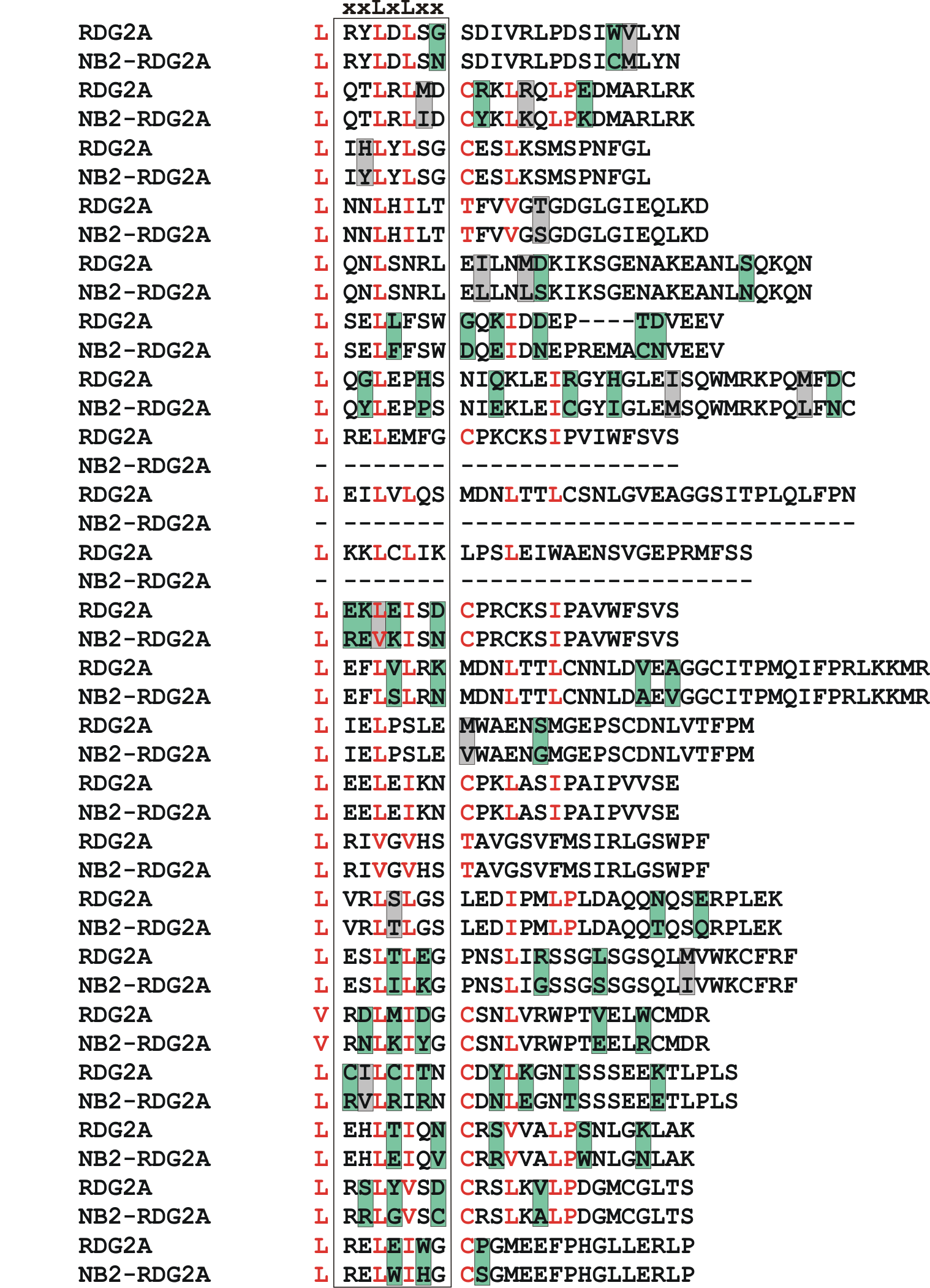

Supplement: Figure S5 — Alignment of the deduced LRR domain sequences of RDG2A and NB2-RDG2A. Substitution differences are boxed; those in grey and green represent conservative and non-conservative substitutions (as defined by ClustalW), respectively. The regions of the LRRs that correspond to the β-strand/β-turn motif xxLxLxx are framed and the leucine (or other aliphatic) residues that form the structural backbone of the LRR units in RDG2A are in red. (9.58 MB TIF) [file pone.0012599.s009.tif]

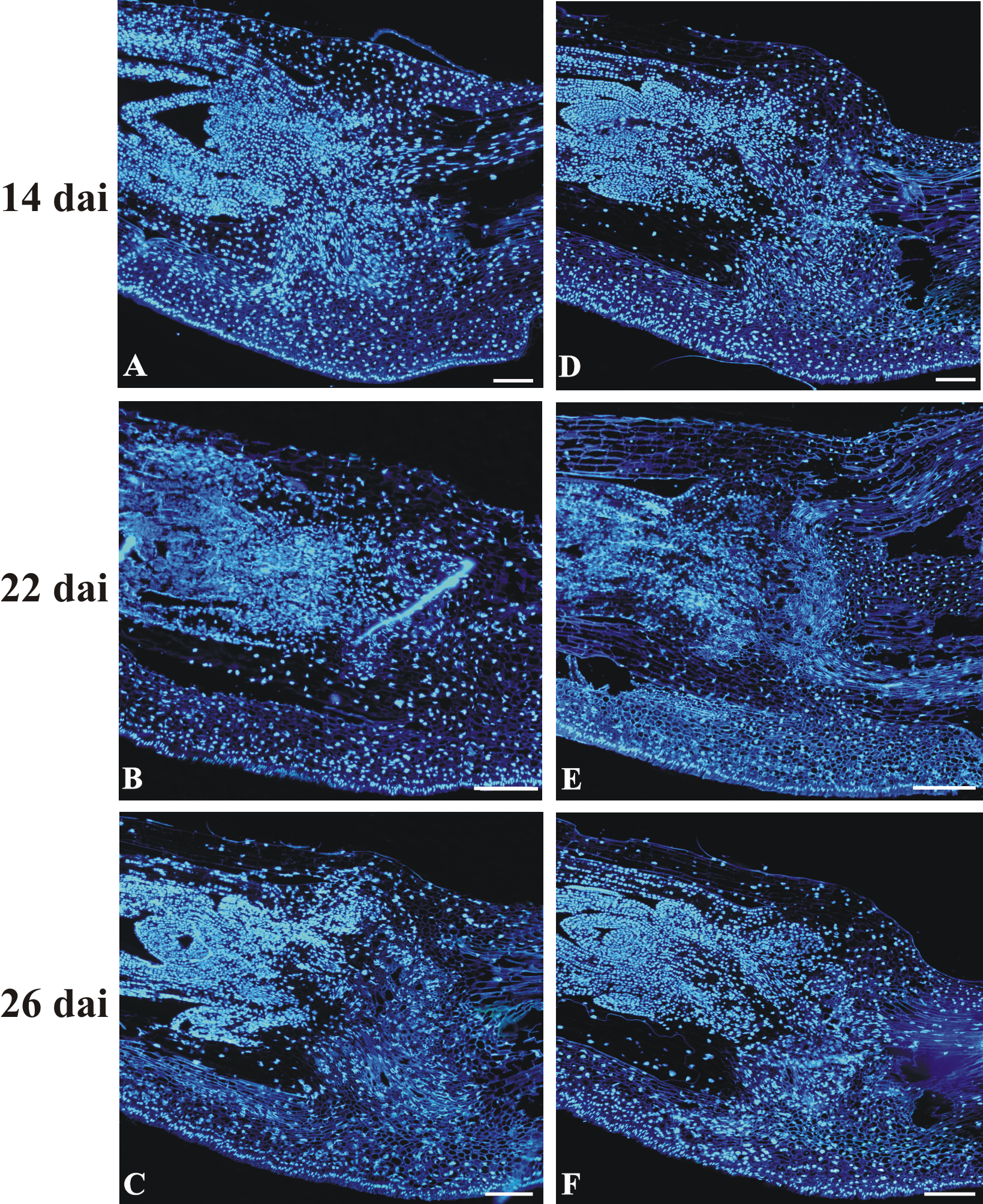

Supplement: Figure S6 — DAPI staining of embryo sections analyzed for autofluorescence and by TUNEL in Figure 7. DAPI staining of nuclei was performed for embryo sections of Figure 7 A and D (A), B and E (B), C and F (C), G and J (D), H and K (E), I and L (F). Scale bars represent 200 µM. (9.88 MB TIF) [file pone.0012599.s010.tif]
