# Supplementary material for: The CC-NB-LRR-Type Rdg2a Resistance Gene Confers Immunity to the Seed-Borne Barley Leaf Stripe Pathogen in the Absence of Hypersensitive Cell Death
Source: PLoS One. 2010 Sep 10;5(9):e12599. doi: 10.1371/journal.pone.0012599 (PMC2937021; doi:10.1371/journal.pone.0012599)
Supplement: Methods S1 — Supplementary text for Materials and Methods. (0.06 MB DOC) [file pone.0012599.s011.doc]

**Methods S1: Supplementary Text for Materials and Methods**

**BAC library screening, cosmid library construction and screening**

Screening of the barley cv. Morex BACs library [1] was performed with the *Rdg2a*-linked molecular marker MWG851, mapped 0.07 cM proximal to *Rdg2a* [2]. A 360 bp fragment of the MWG851 probe sequence was PCR amplified from cv. Thibaut genomic DNA using primers 851-F and 851-R (Table S2), cloned in pGEM-T Easy Vector (Promega), sequenced, and then amplified from the plasmid DNA using the same primer combination to generate the probe template. Hybridization screening of the library was performed as indicated at <http://www.genome.clemson.edu/node/169>, except that sodium phosphate was used at 0.5 M instead of 0.25 M in the hybridization buffer. To identify overlaps, MGW851 hybridizing BAC clones were digested with restriction enzymes *Bam*HI, *Hind*III or *Eco*RI, or the combinations *Bam*HI/*Hind*III, *Bam*HI/*Eco*RI or *Hind*III/*Eco*RI, and the fragments compared by agarose gel electrophoresis. Three clones (146G20, 244G14 and 608H20) were shown to overlap, and were end-sequenced. Ends of clones 244G14 and 608H20 showed similarity to retrotransposon sequences and were not used for marker development. However, both ends of 146G20 were low-copy, enabling the development of the CAPS markers 146.1F-1R and 146.4F-3R. These were respectively mapped 7 cross-overs (CO) distal and two CO proximal to *Rdg2a*, indicating that the clone 146G20 spanned the entire *Rdg2a* locus (Figure 1A). Low-pass shotgun sequencing was conducted by sequencing the ends of 90 BAC-derived plasmid clones. Agarose gel fingerprinting was used to estimate the size of the BACs insert.

For cosmid library preparation, high-molecular-weight DNA was isolated from destarched leaves of the barley cv. Thibaut and partially digested with *Sau*3AI to produce DNA fragments of 30 to 50 kb. After dephosphorylation, the fragments were ligated to the *Xba*I-*Bam*HI linearized SuperCos cosmid vector, according to the manufacturer’s instructions (Stratagene). The ligations were packaged using Gigapack III XL packaging extract (Stratagene) and then used to transform *Escherichia coli* strain XL1-Blue MR (Stratagene). A total of 163 pools averaging 5200 clones each were made and kept frozen as glycerol stocks. The library has an average insert size of 30 kb (range 25 to 45 kb) and represents approximately five genome equivalents. Plasmid DNA was prepared from each pool and was used for PCR screening. Approximately 20,000 clones from each positive pool were screened by hybridization to obtain purified clones.

**Marker analysis and STS mapping**

Isolation of genomic DNA and Southern hybridizations were performed as previously described [2]. Probes for the LRR region of each NB-LRR gene was used for RFLP analysis of *Bam*HI restricted barley genomic DNA. Probes were amplified from cosmid 95-9-3 (for *Nbs1-Rdg2a*) and from cosmid 17-1-1 (for *Nbs2-Rdg2a* and *Nbs3-Rdg2a*) using primers designed on the LRR-encoding and 3’ UTR regions. Amplification products were cloned into the pGEM-T Easy Vector, verified by sequencing and newly amplified from the plasmid DNA using the same primer combination to make the probe template.

PCR markers derived from Morex BACs shotgun sequencing were scored as **previously described [2], using** primers and PCR annealing temperatures shown in Table S2. **Restriction enzymes to be used with these markers were identified using the software “Restriction Enzyme Site Mapper version 3” (**<http://www.restrictionmapper.org/>**) or** dCAPS Finder (<http://helix.wustl.edu/dcaps/dcaps.html>), for **CAPS and** dCAPS markers, respectively**.**

PCR markers to confirm the genomic location of the sequenced Mirco *Nbs1-rdg2a* and *Nbs2-rdg2a* genes were obtained by designing primers flanking insertion/deletion polymorphisms in the putative regulatory regions (Figure S3) - primers Nbs1-14 and Nbs1-19 for *Nbs1-rdg2a* and primers Nbs2-6 and Nbs2-29 for *Nbs2-rdg2a* - and testing for co-segregation of the marker fragments with the *Rdg2a* locus. Primer sequences and PCR annealing temperatures are shown in Table S2.

**Sequencing and sequence analysis**

BAC DNA for sequencing was prepared using the Qiagen Plasmid Midi Kit (Qiagen, Ltd., Crawley, UK) and digested completely with *Hind*III or *Eco*RV. The DNA fragments (1.5 to 3 kb) were cloned into the pBluescript II SK- plasmid vector (Stratagene) and propagated in *E. coli* strain DH10B. DNA from cosmid clones was isolated using the Qiagen Plasmid Midi Kit and digested partially with *Hae*III or *Rsa*I. Fragments between 1 and 3 kb were gel purified, dephosphorylated, extracted once with phenol/chloroform, ethanol precipitated and cloned using the Zero Blunt TOPO PCR Cloning Kit (Invitrogen) according to the manufacturer’s instructions. Plasmid DNA was automatically purified (Biomek 3000, Beckman Coulter) with the Wizard SV96 Plasmid DNA Purification System (Promega). BAC ends and plasmid sequences were obtained on an ABI PRISM 3130xl Genetic Analyzer (Applied Biosystems). Base calling and quality of the shotgun sequences were processed using PHRED [3,4] and assembled using the PHRAP assembly engine (version 0.990329; provided by P. Green, [http://www.phrap.org](http://www.phrap.org/)). The CONSED software package was used to ﬁnish the assembly [5,6]. Gaps between the subcontigs were ﬁlled by direct sequencing of the cosmid clones using 20- to 24-mer oligonucleotides designed at the contig ends. DNA sequences were analyzed using BLASTN, BLASTX, and TBLASTX algorithms [7]. Detailed sequence analyses were performed with the Vector NTI Suite 9 (**InforMax, Invitrogen) and** GAP4 software from the Staden package (<http://staden.sourceforge.net/>, [8]). For gene prediction, the RiceGAAS annotation system [9] was used. Coiled-coil structure was predicted by COILS (<http://www.ch.embnet.org/software/COILS_form.html>). PAML (<http://abacus.gene.ucl.ac.uk/software/paml.html>) was used to determine *Ka/Ks* ratios.

**Reverse-trascription-PCR and rapid amplification of cDNA ends**

Rapid amplification of cDNA ends (RACE) was performed with the GeneRacer kit (Invitrogen, Carlsbad, CA) as recommended by the manufacturer, using 250 ng of DNAse (Ambion, Applied Biosystems) treated poly(A)-RNA obtained from the embryos of NIL3876-*Rdg2a*. RACE and reverse-transcription PCR were carried out both on embryos excised from the seeds at 14 days after inoculation with *P. graminea* isolate Dg2 and on embryos obtained from seeds germinated for 14 days on wet filter paper. Amplifications were performed with Platinum *Pfx* DNA polymerase, using amplification conditions recommended by the manufacturer (Invitrogen). RACE and cDNA products were cloned into the Zero Blunt TOPO vector (Invitrogen) and five independent clones from each amplification were sequenced. To obtain the 5’ end of *Nbs1-Rdg2a*, two rounds of PCR were performed using the gene-specific primer (GSP) nbs1-4 (CAGGCGACAGGTGTTTGTAGCTTA) plus the GeneRacer 5’ primer for the first round amplification, and the GSP nbs1-22 (CGGTGCACATTATCGAGGCG) plus the GeneRacer 5’ nested primer for the second round. 3’ RACE was carried out with GSP nbs1-15 (CAGAACTGCCGCAGTGTAGTAGC) plus the GeneRacer 3’ primer and GSP nbs1-16 (AGCTGAGGAGTCTCTATGTGAGCG) plus the GeneRacer 3’ nested primer. cDNA sequences internal to RACE products were obtained by amplification of three overlapping *Nbs1-Rdg2a* fragments obtained with primer combinations nbs1-9 (ATTGGTCACATGTCGAAGCAAGCAAGTCGC) *plus* D2-17 (TCGCAACTTCCGGCAATCCATTAG), D2-8 (GTTGCTACAGGTATCGGCATCAC) *plus* nbs1-26 (GCTAAACATCCGAGGCTCTCCTACACTA) and nbs1-5 (TAGTGTAGGAGAGCCTCGGATGTT) *plus* nbs1-8 (AGACTCACGCGTATGCCGATTCA).

To obtain the 5’ end of *Nbs2-Rdg2a*, two rounds of PCR were performed, using the GSP nbs2-11 (GCTAGCTTATGGGTTCCAAGGGTCTG) plus the GeneRacer 5’ primer for the first round and the GSP nbs2-12 (GGCTTCGACTTGTGACAACAATGACG) plus the GeneRacer 5’ nested primer for the second round. 3’ RACE was carried out with GSP nbs2-23 (TGTTGCCTCTGGACGCCCAGCAAACC) plus the GeneRacer 3’ primer and GSP nbs2-24 (CAGCTTGATCGGAAGCTCCGGATCG) plus the GeneRacer 3’ nested primer. cDNA sequences internal to RACE products were obtained by amplification of two overlapping *Nbs2-Rdg2a* fragments obtained with primer combinations nbs2-19 (CGGCCTCTATAATGCAGACCCTTGGAA) *plus* nbs2-16 (TTCAACTTGTAACAGTCTATGAGC) and nbs2-20 (GCTCATAGACTGTTACAAGTTGAA) *plus* nbs2-21 (GCAGCCATATATCTTCAGATTTCTC).

To obtain the 5’ end of *Nbs3-Rdg2a*, two rounds of PCR were performed using the GSP nbs3-11 (TCAGGCATGTCACATCTTCCACTTAC) plus the GeneRacer 5’ primer for the first round and the GSP nbs3-12 (CTTCTGTTCTTGCTGGTCCAACAGTTT) plus the GeneRacer 5’ nested primer for the second round. 3’ RACE was carried out with GSP nbs3-43 (GATGTGGGCAGAAAATAGTATGGGAGAG) in combination with GeneRacer 3’ primer and GSP nbs3-42 (AGCATAGTTGGAGCTCACAGTACTGCAGTC) in combination with GeneRacer 3’ nested primer. cDNA sequences internal to RACE products were obtained by amplification of five overlapping Nbs3-Rdg2a fragments obtained with primer combinations nbs3-33 (GCTGAGGAGATGAGCGAGAAGAAGTC) *plus* nbs3-35 (CCAAGATACCTAGCAGACCTCACTGAC), nbs3-36 (AAGAGAGAACAATGGATTTAACACGGAA) *plus* nbs3-25 (GTTGTCAGGTTATCCATCCTCTGTAAGAG), nbs3-30 (CTCTTACAGAGGATGGATAACCTGACAAC) *plus* nbs3-2 (GCATCGTCTTACCAACTCCGGGCAATATTT), nbs3-37 (AGGGGTCTCCGTGTGCTGCACTGC) plus nbs3-38 (GCCATCTCCGGTACCCACAACAA), nbs3-39 (GCTCATGGATTGCCAGAAGTTGCG) *plus* nbs3-40 (GGGTTTCCTCCTCCTCCTCATATGATGAAG).

**RT-PCR and quantitative RT-PCR**

For RT-PCR analysis, total RNA was isolated using Trizol reagent (Invitrogen) from control and inoculated embryos of barley cv. Mirco and NIL3876 at time points 7 and 14 dai. Total RNA was also extracted from the leaves of cvs. Mirco, Thibaut, Golden Promise and transgenic lines grown under inoculated and non-inoculated conditions. Equal amounts of total RNA (10 µg per reaction) were treated with the DNA-free kit (Ambion, Applied Biosystems), and the resulting DNA-free RNA used for first strand cDNA synthesis using an oligo dT primer (Invitrogen). cDNA was diluted 10-fold, quantified by using a Qubit fluorometer (Invitrogen) and 4 ng used as template for RT-PCR in a reaction of 20 µl containing 1× reaction buffer, 2.0 mM MgCl_2_, 0.2 mM of each dNTP, 0.3 µM of each primer, 5% DMSO and 1 U Go *Taq* polymerase (Promega). Cycling conditions were: 2 min at 94°C, followed by 21 cycles (for the barley *Actin* gene; AY145451.1) or 27 cycles (for *Rdg2a* candidates) of 94°C for 40 sec, 60°C for 50 sec, 72°C for 1 min, followed by a final extension step at 72°C for 10 min. PCR cycles were increased to 35 to further test whether genes were expressed in Mirco. Primers are shown in Table S3. Primer combination *Nbs1-Rdg2a* 25-26 was used to detect the endogenous *Rdg2a* gene or *Rdg2a* transgene transcript in transgenic plants (Figure 3a and 3b). Primers used for RT-PCR of the fungal genes *Ubiquitin* and *GTPase activator* were described in [10]. To enable discrimination between the members of the gene family and alleles, RT-PCR primers were designed on two different regions of the LRR-encoding domain where several substitutions or small insertion/deletions between the genes were present. The amplicons were then sequenced to confirm specific amplification for each *Rdg2a* candidate and allele.

Quantitative real-time PCR (qRT-PCR) was performed using total RNA isolated from control and inoculated embryos of NIL3876 at 7, 14, 18, 22 and 28 dai. Two independent biological replicates were carried out for each time point and inoculation/control treatment. RNA treatments and first strand cDNA synthesis and quantification were carried out as described for the RT-PCR experiments. qRT-PCR reactions conditions and data analysis were as previously described [10]. The qRT PCR data were plotted as ΔR_n_ fluorescence signal versus cycle number. The SDS 7300 absolute quantification software (Applied Biosystems) calculates the ΔR_n_ using the equation ΔRn = (R_n_^+^) – (R_n_^-^), where R_n_^+^ is the fluorescence signal of the product at any given time and R_n_^-^ is the fluorescence signal of the baseline emission during cycles 6 to 13. An arbitrary threshold was set at midpoint of the log ΔRn versus cycle number at which the ΔRn crosses the threshold (C_t_). The C_t_ was used to calculate the fold changes (FC) in each infected sample with respect to the expression level detected in corresponding sample in control conditions at same time point (baseline) with the following formula:

FC= 2^-ΔΔCt^, where ΔΔC_t_ = (C_t_ _target_-C_t_ _βact_)_infected_ _sample_-(C_t_ _target_-C_t_ _βact_)_uninfected sample_

Normalization was carried out with the β-actin constitutively expressed gene. Four to six replicates RT-PCR were performed for each of the two independent inoculations. SD was calculated across all RT-PCR replicates (four to six from each of two independent inoculations). For the *Nbs2-Rdg2a* gene, a Wilcoxon two group test [11,12] was used to analyze the ΔCt values (Ct target-Ct βact) in infected and un-infected samples at each time point of inoculation. Data from two biological replicates with four technical replicates each were used for the analysis. In all the time points where an increased transcription of the gene in response to pathogen inoculation was observed (7, 14 and 18 dai) the test yielded *P*-Values <0.05, indicating that ΔΔCt was significantly different from 0 and that there was a significant effect. No statistically different ΔΔCt values were observed for samples collected at 22 dai and 28 dai. The Wilcoxon two group test was also applied to verify statistic significance of the *Rdg2a* and *Nbs2-Rdg2a* transcription rates.

Primer pairs used for the analysis (nbs1-27 plus nbs1-28 for *Nbs1-Rdg2a* and nbs2-2 plus nbs2-5 for *Nbs2-Rdg2a*) were designed using the software Vector NTI Suite 9 (Informax, Invitrogen) and are shown in Table S3.

**Phylogenetic analysis**

Amino acid sequence alignments were conducted with the CLUSTALX program [13]. A phylogenetic tree was produced using the Neighbour-Joining (NJ) method, with the *Arabidopsis thaliana* RPM1 protein being used as the outgroup. The degree of branching was determined within the NJ framework using the bootstrap procedure [14]. The original data set was re-sampled 1000 times. Bootstrap values are indicated as percentages.

**References**

1. Yu Y, Tokins JP, Waugh R, Frisch DA, Kudrna D, et al. (2000). A bacterial artificial chromosome library for barley (*Hordeum vulgare L.*, ) and the identification of clones containing putative resistance genes. Theor Appl Genet 101: 1093-1099.

2. Bulgarelli D, Collins NC, Tacconi G, Dall’Aglio E, Brueggeman R, et al. (2004) High-resolution genetic mapping of the leaf stripe resistance gene *Rdg2a* in barley. Theor Appl Genet 108: 1401-1408.

3. Ewing B, Green P (1998) Base-calling of automated sequencer taces using phred. II. Error probabilities. Genome Res 8: 186–194.

4. Ewing B, Hillier L, Wendl MC, Green P (1998) Base-calling of automated sequencer traces using phred. I. Accuracy assessment. Genome Res 8: 175–185.

5. Gordon D, Abajian C, Green P (1998) Consed: a graphical tool for sequence finishing. Genome Res 8: 195-202.

6. Gordon D, Abajian C, Green P (2001) Automated finishing with Autofinish. Genome Res 11: 614-625.

7. Altschul SF, Madden TL, Schäffer AA, Zhang J, Zhang Z, et al. (1997) Gapped BLAST and PSI-BLAST: a new generation of protein database search programs. Nucleic Acids Res 25: 3389–3402.

8. Staden R, Beal KF, Bonfield JK (1998) The Staden Package, 1998. Computer Methods in Molecular Biology 132: 115-130.

9. Sakata K, Nagamura Y, Numa H, Antonio BA, Nagasaki H, et al. (2002). RiceGAAS: An automated annotation system and database for rice genome sequence. Nucleic Acids Res 30: 98–102.

10. Haegi A, Bonardi V, Dall’Aglio E, Glissant D, Tumino G, et al. (2008) Histological and molecular analysis of Rdg2a barley resistance to leaf stripe. Mol Plant Pathol 9: 463-478.

11. Hollander M, Wolfe DA (1973) Nonparametric Statistical Methods. John Wiley and Sons, New York, 503.

12. Yuan JS, Reed A, Chen F, Stewart Jr CN (2006) Statistical analysis of real time PCR data. BMC Bioinformatics 7: 85.

13. Thompson JD, Gibson TJ, Plewniak F, Jeanmougin F, Higgins DG (1997) The CLUSTAL_X windows interface: flexible strategies for multiple sequence alignment aided by quality analysis tools. Nucleic Acids Res 25: 4876–4882.

14. Felsenstein J (1985) Confidence limits on phylogenesis – an approach using the bootstrap. Evolution 39: 783-791.
